# The antioxidant and DNA-repair enzyme apurinic/apyrimidinic endonuclease 1 limits the development of tubulointerstitial fibrosis partly by modulating the immune system

**DOI:** 10.1038/s41598-019-44241-z

**Published:** 2019-05-24

**Authors:** Keisuke Maruyama, Naoki Nakagawa, Tatsuya Aonuma, Yukihiro Saito, Taiki Hayasaka, Kohei Kano, Kiwamu Horiuchi, Naofumi Takehara, Jun-ichi Kawabe, Naoyuki Hasebe

**Affiliations:** 1 Division of Cardiology, Nephrology, Respiratory and Neurology, Department of Internal Medicine, Asahikawa Medical University, Asahikawa, Japan; 2 Center for Advanced Research and Education, Asahikawa Medical University, Asahikawa, Japan; 3 Department of Cardiovascular Regeneration and Innovation, Asahikawa Medical University, Asahikawa, Japan

**Keywords:** Computational biology and bioinformatics, Kidney diseases

## Abstract

Apurinic/apyrimidinic endonuclease 1 (APE1) is a multifunctional protein that controls the cellular response to oxidative stress and possesses DNA-repair functions. It has important roles in the progression and outcomes of various diseases; however, its function and therapeutic prospects with respect to kidney injury are unknown. To study this, we activated APE1 during kidney injury by constructing an expression vector (pCAG-APE1), using an EGFP expression plasmid (pCAG-EGFP) as a control. We performed unilateral ureteral obstruction (UUO) as a model of tubulointerstitial fibrosis on ICR mice before each vector was administrated via retrograde renal vein injection. In this model, pCAG-APE1 injection did not produce any adverse effects and significantly reduced histological end points including fibrosis, inflammation, tubular injury, and oxidative stress, as compared to those parameters after pCAG-EGFP injection. qPCR analysis showed significantly lower expression of *Casp3* and inflammation-related genes in pCAG-APE1-injected animals compared to those in pCAG-EGFP-injected UUO kidneys. RNA-Seq analyses showed that the major transcriptional changes in pCAG-APE1-injected UUO kidneys were related to immune system processes, metabolic processes, catalytic activity, and apoptosis, leading to normal kidney repair. Therefore, APE1 suppressed renal fibrosis, not only via antioxidant and DNA-repair functions, but also partly by modulating the immune system through multiple pathways including *Il6*, *Tnf*, and chemokine families. Thus, therapeutic APE1 modulation might be beneficial for the treatment of renal diseases.

## Introduction

Chronic kidney disease (CKD) is a worldwide public health issue because patients with this condition are likely to progress to end stage renal disease (ESRD), in addition to suffering from cardiovascular disease^1,2^. Tubulointerstitial fibrosis is a chronic and progressive process affecting the kidneys during aging and in CKD, regardless of the cause^3^. However, at present, there are no specific treatment options to slow renal fibrosis for most patients with CKD.

Apurinic/apyrimidinic endonuclease 1 (APE1) is a multifunctional protein that controls cellular responses to oxidative stress, in addition to possessing DNA-repair functions^4,5^. APE1 has important roles in the progression and outcomes of various diseases. Specifically, we previously reported that APE1 enhances vascular repair *in vivo* by promoting endothelial progenitor cell adhesion^6^, and that APE1- overexpressing cardiac progenitor cells can survive and function in the ischemic heart, restoring cardiac function and suppressing cardiac inflammation and fibrosis^7^. Previous reports showed that APE1 expression is associated with the expression of apoptosis-related proteins including p53 and Caspase-3 in diabetic nephropathy^8^, unilateral ureteral obstruction (UUO)^9^, and aged kidney^10^ models and that APE1 has a pro-fibrotic effect on proximal tubular epithelial cells in a redox-dependent manner^9^. However, to date, no studies have indicated how activated APE1 affects kidney disease progression and outcome *in vivo*.

The present study investigated the therapeutic prospects of exogenous APE1 in a mouse model of UUO by increasing its expression using a plasmid vector. To elucidate the in-depth mechanism associated with this effect, bioinformatics analyses were performed based on Gene Ontology (GO) and the Kyoto Encyclopedia of Genes and Genomes (KEGG) database^11–13^.

## Results

### Renal expression of APE1 mRNA and protein is increased significantly after UUO

**Figure 1:**
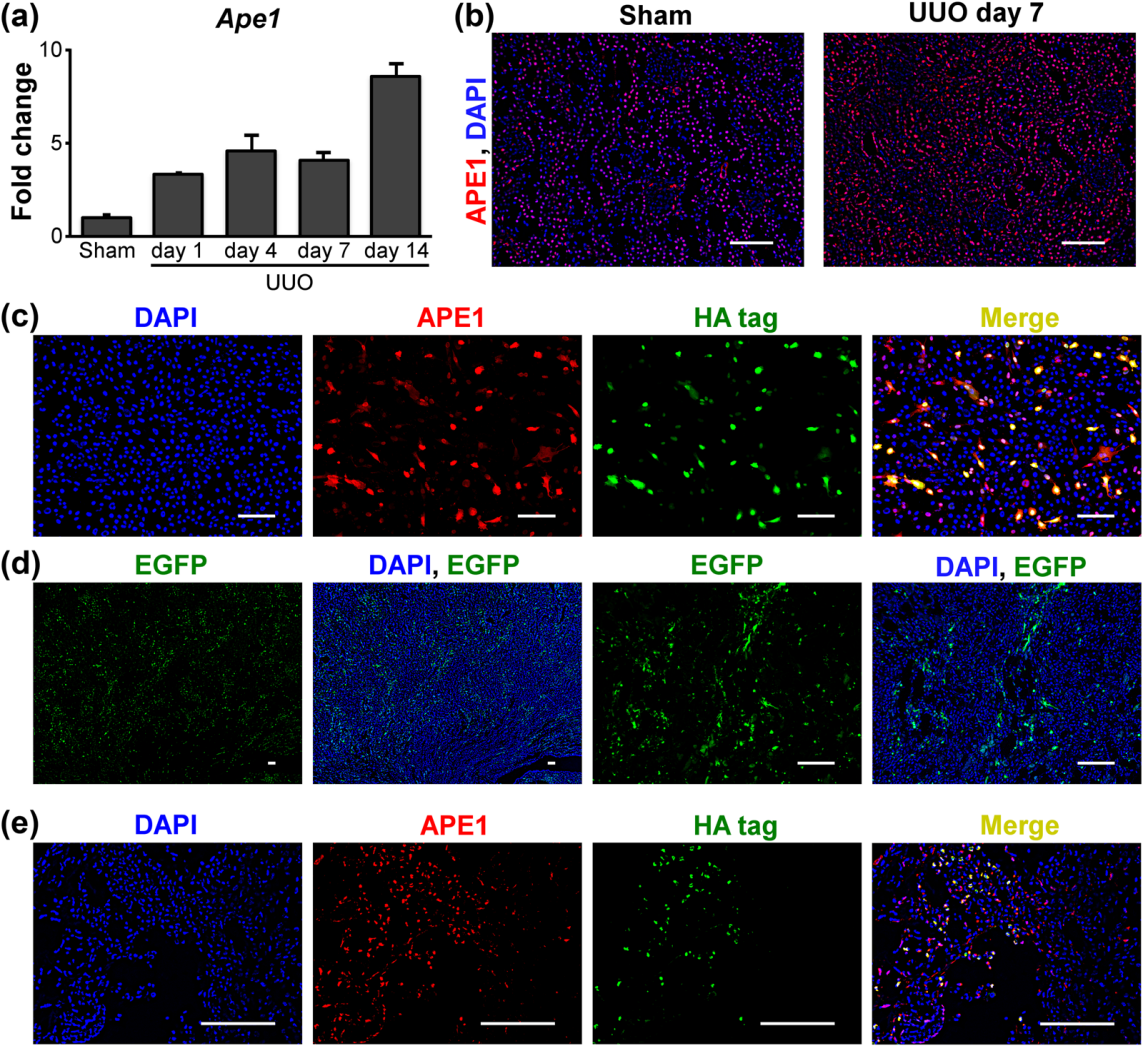
APE1 expression in the kidney of sham-operated and unilateral obstructed ureter (UUO) mice and functional analysis of plasmid vectors *in vitro* and *in vivo*. (**a**) *APE1* mRNA expression in the control and ureter-obstructed kidney (n = 3/group). Data are presented as the mean ± SEM. (**b**) Immunohistological expression of APE1 in the control and UUO kidney. APE1 exhibited nuclear localization in essentially all cells of sham kidneys, whereas in UUO kidneys, APE1 staining was mainly observed in tubular epithelial cells and interstitial cells. (**c**) Transgene expression of pCAG-APE1 vector in BHK-21 cells. (**d**) EGFP expression in the pCAG-EGFP-injected interstitium 4 days after UUO. (**e**) APE1 expression in the pCAG-APE1-injected interstitium 4 days after UUO. Scale bars: 100 μm.

We first investigated whether the kidney responds to stress induced by UUO by modulating APE1 expression. For this, we examined the expression of *APE1* mRNA. These levels increased significantly in the ureter-obstructed kidney on day 1 post-operation compared to those in control kidneys, and this increase in expression over control levels remained constant on days 4 and 7 post-UUO (Fig. 1a). A further significant increase in the expression of *APE1* was found in ureter-obstructed kidneys on day 14 post-operation (Fig. 1a), suggesting that APE1 might play a role in the pathogenesis of tubulointerstitial fibrosis after UUO.

Given the accumulating evidence suggesting that oxidative stress plays a role in the development of kidney damage after ureteral obstruction and the central role for APE1 in the repair of DNA lesions induced by oxidative stress, we next analyzed the localization of APE1 in kidney tissues. Immunohistological staining revealed that APE1 exhibited nuclear localization in essentially all cells of sham kidneys (Fig. 1b), in accordance with its function in DNA repair. In contrast, APE1 staining was observed especially in tubular epithelial cells and interstitial cells of ureter-obstructed kidneys on day 7 post-UUO (Fig. 1b), consistent with APE1 up-regulation at this time point.

### Localization of transgene expression *in vitro* and *in vivo*

Next, we tested transgene expression of pCAG-APE1 or pCAG-EGFP in BHK-21 cells after polyethyleneimine transfection. Specific fluorescence was mainly distributed in the nuclei of BHK-21 cells transfected with pCAG-APE1 (Fig. 1c) and pCAG-EGFP (data not shown).

To clarify the transgene expression site *in vivo*, we delivered 200 μg of pCAG-APE1 or pCAG-EGFP into the kidneys and immunostained kidney sections on day 4 post-UUO. The interstitium in the cortex of pCAG-EGFP-injected kidneys showed positive staining (Fig. 1d). Moreover, in this tissue from pCAG-APE1-injected kidneys, APE1 was found to co-localize with the HA-tag (Fig. 1e). These analyses showed that the transgene was expressed in interstitial fibroblasts as previously reported^14,15^.

### Tubulointerstitial fibrosis, inflammation, tubular injury, and oxidative stress in UUO kidneys are suppressed by *APE1* transfection

**Figure 2:**
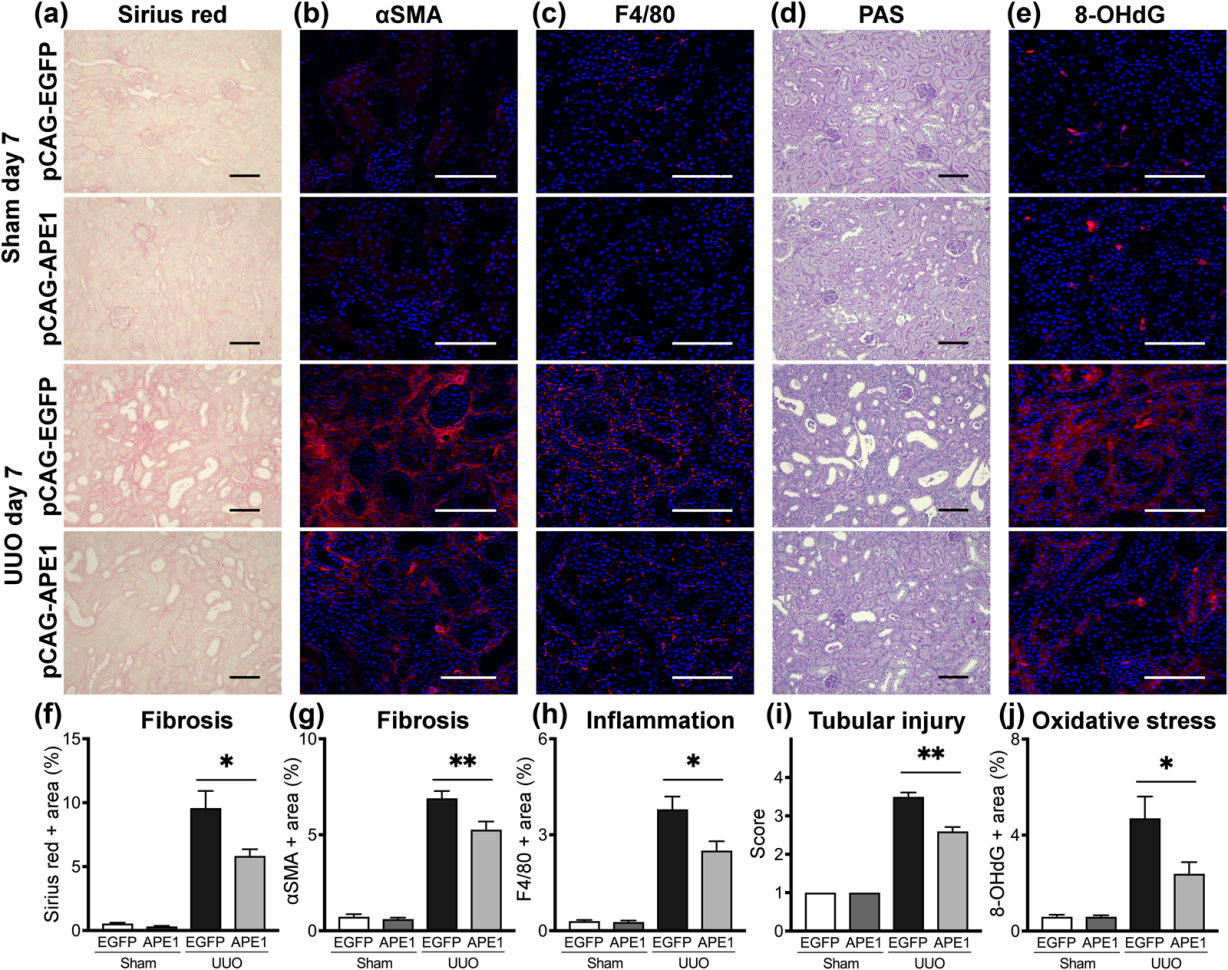
pCAG-APE1 injection suppresses interstitial fibrosis, inflammation, and oxidative stress. Mice were subjected to unilateral obstructed ureter (UUO) or sham procedures and injected with pCAG-APE1 or pCAG-EGFP (control) vectors. (**a**) Sirius red-stained images of kidney cortex. (**b**) Images of αSMA-positive cells in the kidney cortex. (**c**) Images of F4/80-positive cells in the kidney cortex. (**d**) Images of periodic acid–Schiff (PAS) staining in the kidney cortex. (**e)** Images of 8-hydroxy-2′-deoxyguanosine (8-OHdG)-positive cells in the kidney cortex. (**f–j**) Quantification of fibrosis based on Sirius red staining, myofibroblasts detected by αSMA, inflammation identified by F4/80 expression, tubular injury based on PAS staining, and oxidative stress detected by 8-OHdG. Scale bars: 100 μm. n = 5–6/group. *P < 0.05, **P < 0.01 (ANOVA with Tukey’s post hoc analysis). Data are presented as the mean ± SEM.

**Figure 3:**
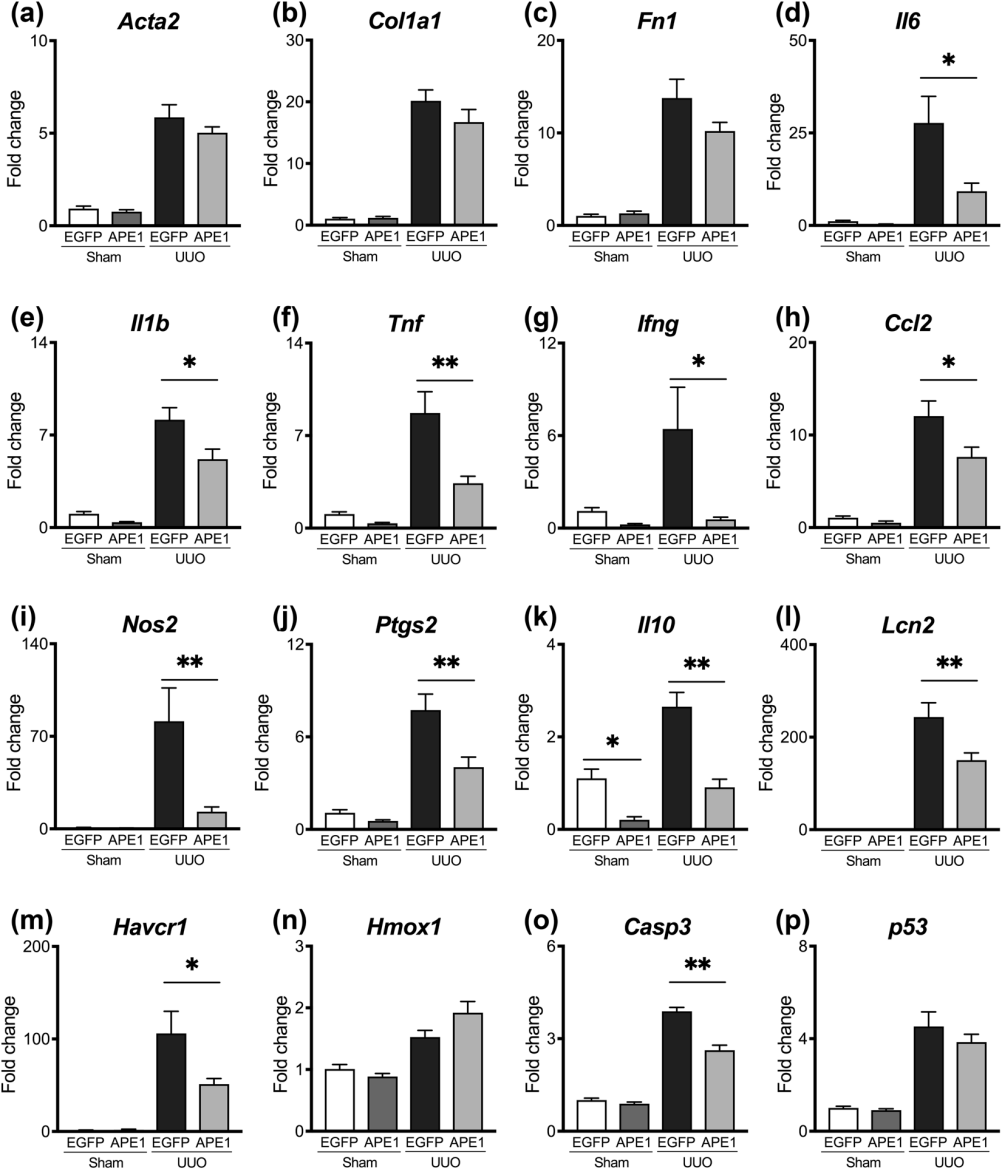
pCAG-APE1 injection reduces the expression of mRNA associated with inflammation and apoptosis. qPCR measuring markers of fibrosis (*Acta2* (**a**)*, Col1a1* (**b**)*, Fn1* (**c**)), inflammation (*Il6* (**d**), *Il1b* (**e**), *Tnf* (**f**), *Ifng* (g), *Ccl2* (**h**), *Nos2* (**i**), *Ptgs2* (**j**)*, Il10* (**k**)), tubular injury (*Lcn2 [Ngal]* (**l**)*, Havcr1 [Kim1]* (**m**)), anti-oxidation *(Hmox1* (**n**)), and apoptosis (*Casp3* (**o**)*, p53* (**p**)), comparing mice subjected to unilateral obstructed ureter (UUO) procedures and sham-operated mice, injected with pCAG-APE1 or pCAG-EGFP (control) vectors. Data are presented as the mean ± SEM.

To understand the effect of *APE1* transfection on kidney disease progression, histological parameters were evaluated in samples harvested 7 days after sham or UUO procedures. pCAG-EGFP-injected mice exhibited significant tubulointerstitial pathology in UUO kidneys, characterized by interstitial fibrosis, myofibroblast accumulation, inflammation, and tubular injury (Fig. 2a–d,f–i), whereas there were no significant differences between pCAG-EGFP- (control) and pCAG-APE1-injected sham mice on day 7. However, the extent of interstitial fibrosis was markedly attenuated by pCAG-APE1 transfection in UUO mice (Fig. 2a,f). Consistent with the reduction in fibrosis, myofibroblasts, as detected by αSMA staining, were significantly reduced after pCAG-APE1 transfection (Fig. 2b,g). This was supported by a parallel decrease in *Acta2* gene transcripts, which encodes αSMA, and fibrillar collagen-encoding genes such as *Col1a1* and *Fn1* (Fig. 3a–c), although these differences were not significant. Further, inflammatory F4/80^+^ macrophages were significantly reduced after pCAG-APE1 transfection (Fig. 2c,h), with a parallel marked decrease in inflammatory gene transcripts such as *Il6, Il1b*, *Tnf*, *Ifng*, *Ccl2*, *Nos2*, and *Ptgs2* (Fig. 3d–j), whereas the anti-inflammatory cytokine *Il10* was also significantly reduced after pCAG-APE1 transfection (Fig. 3k).

Next, we analyzed the tubular injury, which is also a hallmark of CKD. The extent of tubular injury in the renal cortex was obvious in pCAG-EGFP-injected UUO kidneys (Fig. 2d,i), whereas the histological change and the mRNA expression levels of neutrophil gelatinase–associated lipocalin (*Ngal*, *Lcn2*) and Kim1 (*Havcr1*) were significantly reduced after pCAG-APE1 transfection (Figs 2d,i and 3l,m). Furthermore, the cytoplasmic and nuclear accumulation of oxidative stress, as detected by 8-OHdG, which is a product and biomarker of oxidative DNA damage, in UUO kidneys was significantly reduced by pCAG-APE1 transfection (Fig. 2e,j). This was supported by an increase in *Hmox1* gene transcripts, which encodes heme oxygenase 1, an important defensive mechanism against oxidative stress^16^ (Fig. 3n). Consistent with the reduction in oxidative stress, the *Casp3* gene transcript, which encodes the Caspase-3, a marker of oxidative stress-induced apoptosis, was significantly suppressed by pCAG-APE1 transfection (Fig. 3o). There were no significant differences in *p53* between pCAG-EGFP- and pCAG-APE1-injected UUO kidneys (Fig. 3p). Overall these results suggest that APE1 transfection reduces fibrogenesis, tubular injury, and inflammation, as well as the accumulation of oxidative stress, via the central role of this protein in DNA repair.

### Bioinformatics analyses show that APE1 modulates the immune system and metabolic processes in UUO kidneys

To gain deeper insight into the role of APE1 in modulating the fibrotic process, we assessed the effects of APE1 transfection on global gene expression during the development of tubulointerstitial fibrosis. On day 7, principal component analysis (PCA) revealed profound changes in the transcriptional profiles of pCAG-EGFP-injected mice relative to those of sham-operated mice (Supplementary Fig. 1). pCAG-APE1 injection partly reversed their position along the principal axes, implying that therapy reduced the deviation from a healthy homeostatic state through mechanisms that are not entirely understood. Therefore, we applied bioinformatics analyses based on differential expression, Gene Ontology (GO) analyses, and the Kyoto Encyclopedia of Genes and Genomes (KEGG) database to identify pathways associated with coordinately regulated genes that are activated or suppressed during the progression of kidney disease, and to determine whether these processes are regulated by APE1.

**Figure 4:**
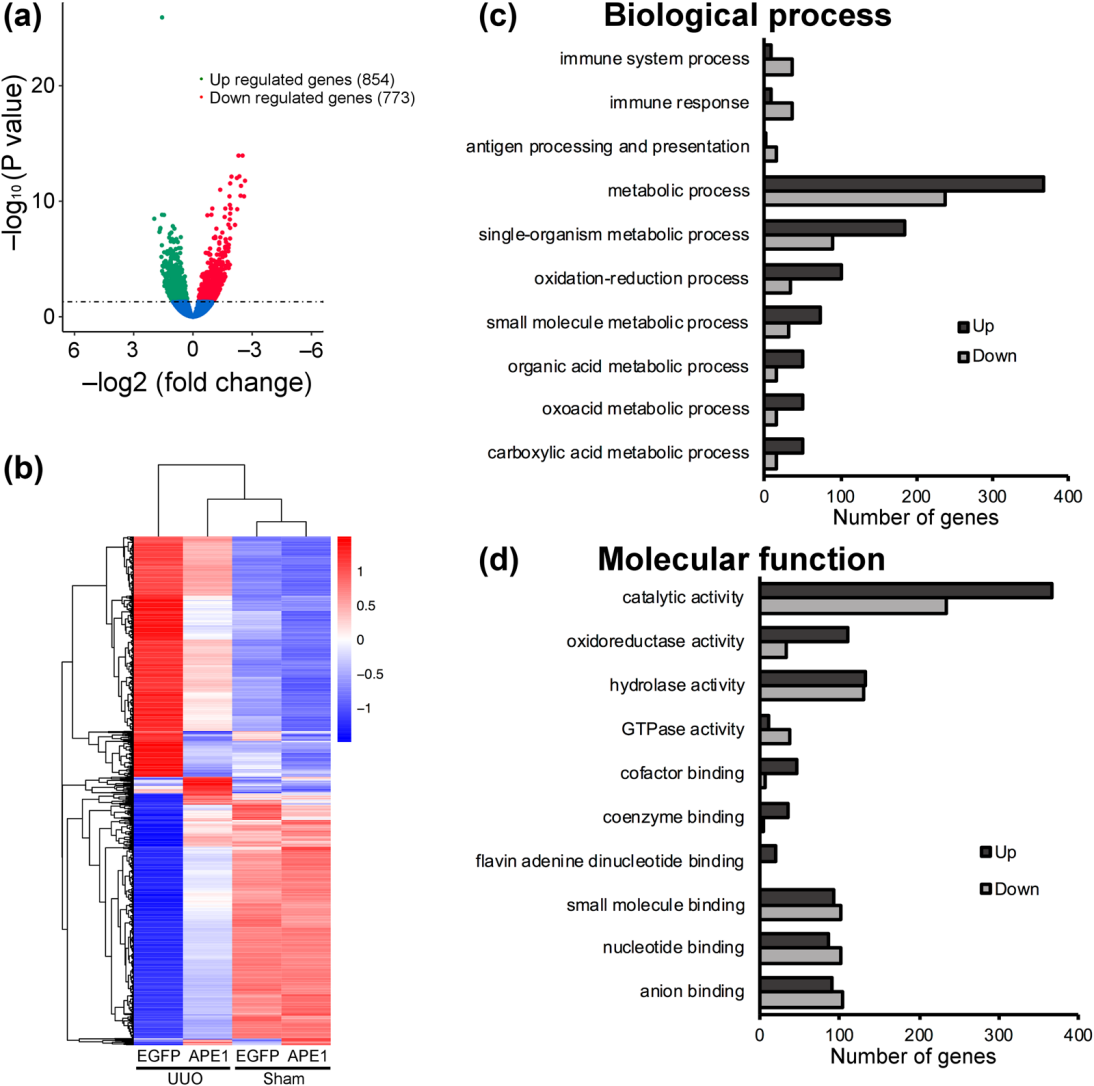
Differentially-expressed gene (DEG) results and the 10 most enriched Gene Ontology (GO) terms comparing pCAG-APE1- and pCAG-EGFP-injected unilateral obstructed ureter (UUO) kidneys. (**a**) Volcanic plot of DEGs. Red dots represent significantly up-regulated DEGs (n = 854) and green dots represent significantly down-regulated genes (n = 773). (**b**) Hierarchical clustering heatmap of DEGs across four comparisons. Red represents highly-expressed genes and blue represents low gene expression. Color descending from red to blue indicates log _10_(FPKM + 1) from high to low expression. (**c,d**) The histogram shows the topological relationships among the 10 most enriched GO terms. Circles and boxes with different colors represent enriched GO terms based on biological process (**c**) and molecular function (**d**) categories.

Differential expression analysis was performed using the DESeq2 R package. A volcanic plot of significantly differentially expressed genes was generated; the red dots represent significantly up-regulated genes (n = 854), whereas the green dots represent significantly down-regulated genes (n = 773) after pCAG-APE1 transfection (Fig. 4a). A heatmap showed that pCAG-APE1-injected UUO kidneys exhibited markedly differential gene expression patterns compared to those of pCAG-EGFP-injected UUO kidneys (Fig. 4b).

**Figure 5:**
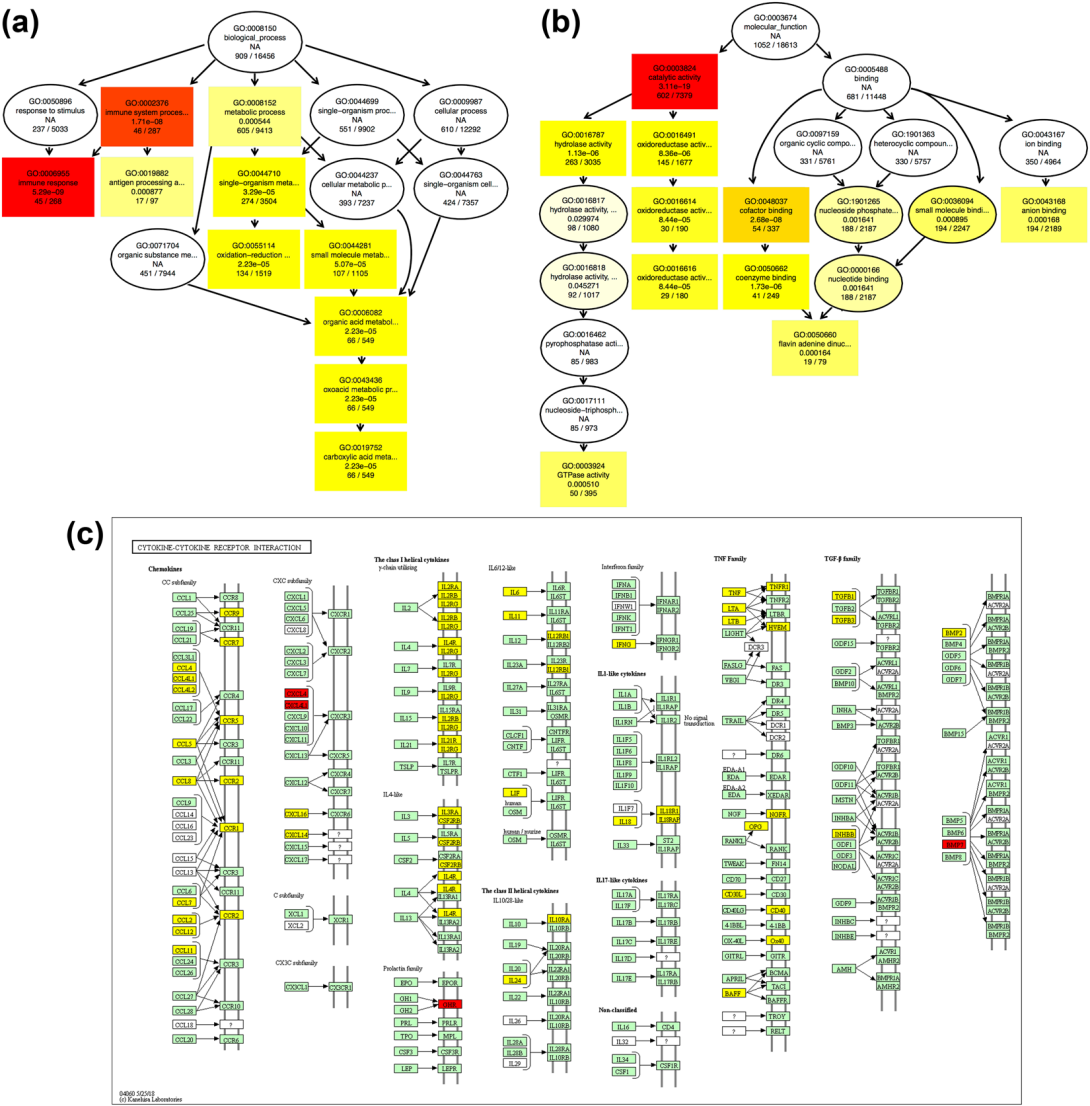
Directed acyclic graph (DAG) of the 10 most enriched Gene Ontology (GO) terms and cytokine–cytokine receptor interaction pathways based on KEGG analysis. pCAG-APE1- and pCAG-EGFP-injected unilateral obstructed ureter (UUO) kidneys were compared for differentially-expressed genes. The DAG shows the topological relationships among the 10 most enriched GO terms. Circles and boxes with different colors represent enriched GO terms for biological process and molecular function categories. Every node indicates one GO term; boxes represent the top 10 enriched GO terms and the P value of the enrichment analysis. The enrichment degree is illustrated by color shades, with the darker the shade, the higher the enrichment. (**a**) The DAG of the 10 most enriched GO terms in the biological process category. Note that immune response was the most enriched GO term among biological processes. (**b**) The DAG of the 10 most enriched terms for molecular function. Note that catalytic activity was the most enriched GO term among molecular functions. (**c**) Cytokine–cytokine receptor interaction pathways based on KEGG analysis (KEG map04060)^11–13^. Yellow rectangles represent down-regulated genes, whereas red rectangles represent up-regulated genes after pCAG-APE1 injection. Copyright permission of this KEGG pathway map was kindly provided by the Kanehisa laboratory.

To further understand the function of differentially-expressed genes (DEGs), GO term enrichment analysis (q < 0.05) was performed. A GO enrichment histogram intuitively shows the number of genes associated with a term, distributed across biological processes and molecular functions. The histogram shows the top 10 enriched GO biological process terms (Fig. 4c) and molecular function terms (Fig. 4d). The most frequent GO subcategories for DEGs were metabolic process and catalytic activity. A Directed acyclic graph (DAG) was used for the graphical representation of GO enrichment analysis based on APE1-target genes. The GO terms shown in red indicate significant differences in immune system processes and immune responses, based on biological process terms (Fig. 5a), and in catalytic activity, based on molecular function terms (Fig. 5b). Within the immune response category, we found that *Il6*, *Tnf*, *Ifng*, and various chemokine families in the immune response were markedly down-regulated after pCAG-APE1 transfection (Table 1), whereas within the catalytic activity and metabolic process term, *Nos2*, *Ptgs2*, *Casp1*, *Casp4*, *Casp7*, *Casp12*, *Jak2*, and *Jak3* were also markedly down-regulated (Supplementary Tables 1, 2). These results suggest that the protective effects of APE1 are mediated by DNA repair activity, in addition to fibrosis-promoting effects due to its role in transcriptional regulation.

**Table 1:** Significant differentially expression genes of immune response between pCAG-EGFP (control) and pCAG-APE1 injected kidneys after UUO.

ID	Gene	EGFP_readcount	APE1_readcount	log2(fold change)	P value	Regulation
ENSMUSG00000058914	C1qtnf3	670.05	1572.18	1.23	2.49E-05	Up
ENSMUSG00000006342	Susd2	1983.90	3818.21	0.94	3.31E-05	Up
ENSMUSG00000019989	Enpp3	1147.16	1801.11	0.65	0.0029113	Up
ENSMUSG00000024766	Lipo1	682.28	1108.97	0.70	0.0050383	Up
ENSMUSG00000029373	Pf4	64.11	126.74	0.98	0.010236	Up
ENSMUSG00000063683	Glyat	3673.57	5958.70	0.70	0.011285	Up
ENSMUSG00000042428	Mgat3	1368.12	2345.47	0.78	0.028499	Up
ENSMUSG00000003420	Fcgrt	2567.23	3847.36	0.58	0.03789	Up
ENSMUSG00000032357	Tinag	1779.99	2428.05	0.45	0.048384	Up
ENSMUSG00000079507	H2-Q1	211.26	15.39	−3.78	1.68E-12	Down
ENSMUSG00000035352	Ccl12	519.86	178.62	−1.54	1.33E-07	Down
ENSMUSG00000021508	Cxcl14	1404.26	628.75	−1.16	1.53E-07	Down
ENSMUSG00000036905	C1qb	3689.92	2232.91	−0.72	3.04E-06	Down
ENSMUSG00000016283	H2-M2	495.02	137.11	−1.85	1.89E-05	Down
ENSMUSG00000028776	Tinagl1	9626.17	4349.81	−1.15	3.54E-05	Down
ENSMUSG00000016206	H2-M3	727.75	264.35	−1.46	0.0001482	Down
ENSMUSG00000024399	Ltb	533.11	223.21	−1.26	0.0001539	Down
ENSMUSG00000035929	H2-Q4	4619.43	1961.31	−1.24	0.0001688	Down
ENSMUSG00000055413	H2-Q5	95.99	39.78	−1.27	0.000446	Down
ENSMUSG00000067341	H2-Eb2	153.37	34.53	−2.15	0.0005087	Down
ENSMUSG00000055170	Ifng	62.15	2.47	−4.66	0.000546	Down
ENSMUSG00000036887	C1qa	5574.69	3432.94	−0.70	0.0005611	Down
ENSMUSG00000060550	H2-Q7	3813.56	1544.61	−1.30	0.0010286	Down
ENSMUSG00000025746	Il6	62.82	18.10	−1.80	0.0010393	Down
ENSMUSG00000073409	H2-Q6	2692.64	1130.14	−1.25	0.0012945	Down
ENSMUSG00000018930	Ccl4	57.16	14.08	−2.02	0.0017748	Down
ENSMUSG00000061232	H2-K1	41864.71	18378.29	−1.19	0.0024271	Down
ENSMUSG00000034394	Lif	1110.57	504.50	−1.14	0.003794	Down
ENSMUSG00000035042	Ccl5	3077.49	1184.86	−1.38	0.0038083	Down
ENSMUSG00000024401	Tnf	199.32	69.76	−1.51	0.0048196	Down
ENSMUSG00000026395	Ptprc	2145.18	986.01	−1.12	0.0075049	Down
ENSMUSG00000024402	Lta	38.65	7.28	−2.41	0.0082132	Down
ENSMUSG00000060183	Cxcl11	2203.65	249.20	−3.14	0.011779	Down
ENSMUSG00000009185	Ccl8	461.51	189.32	−1.29	0.014702	Down
ENSMUSG00000073412	Lst1	334.81	156.15	−1.10	0.015986	Down
ENSMUSG00000028362	Tnfsf8	131.45	59.66	−1.14	0.017298	Down
ENSMUSG00000035373	Ccl7	316.38	196.28	−0.69	0.017357	Down
ENSMUSG00000024610	Cd74	78002.64	47007.99	−0.73	0.020321	Down
ENSMUSG00000020676	Ccl11	76.72	21.64	−1.83	0.021359	Down
ENSMUSG00000079505	Gm11131	71.32	27.81	−1.36	0.025361	Down
ENSMUSG00000067203	H2-K2	874.83	409.19	−1.10	0.027143	Down
ENSMUSG00000041538	H2-Ob	222.78	109.10	−1.03	0.027458	Down
ENSMUSG00000031497	Tnfsf13b	95.32	53.40	−0.84	0.039935	Down
ENSMUSG00000079491	H2-T10	1309.73	728.29	−0.85	0.045468	Down
ENSMUSG00000036322	H2-Ea-ps	366.32	161.89	−1.18	0.047712	Down

To identify APE1-target genes associated with immune responses during the development of renal fibrosis, we performed KEGG enrichment analysis^11–13^. This showed that various cytokine and cytokine receptors were enriched within the regulation of immune responses category. The most highly enriched pathway was cytokine–cytokine receptor interactions, which included *Il6*, *Tnf*, *Ifng*, *Tgfb1*, and various chemokine families including *Ccl2* (monocyte chemoattractant protein-1: MCP-1) and *Ccl5* (regulated upon activation, normal T cell expressed and secreted: RANTES) (Fig. 5c), suggesting that APE1 limits the development of tubulointerstitial fibrosis partly by modulating multiple pathways associated with the immune system.

## Discussion

This report demonstrates that APE1 transfection using retrograde renal vein injection limits the development of tubulointerstitial fibrosis not only via DNA-repair functions but also partly by modulating the immune system through different pathways including *Il6*, *Tnf*, *Ifng*, *Tgfb1*, and various chemokine families including MCP-1 and RANTES. APE1 thus represents a potential target for chronic inflammatory diseases associated with fibrosis.

Suppressing the development of tubulointerstitial fibrosis has emerged as an important strategy to manage chronic kidney diseases because it determines disease prognosis, and there are few anti-fibrotic therapies that can prevent the progression of CKD to ESRD^17^. Therefore, extensive research has been conducted to clarify the mechanism associated with tubulointerstitial fibrosis, and many signaling molecules have been proposed to participate in the pathogenesis of this condition. These signaling molecules including chemokines, inflammatory cytokines, and profibrotic growth factors mediate the sequential events that take place during the development of tubulointerstitial fibrosis^18,19^. However, the role of APE1, as a key molecule in this process, was previously not determined.

Although APE1 has been reported to control cellular responses to oxidative stress and mediate DNA-repair functions^4,5^, this report sheds light on additional APE1 homeostatic functions. First, the renal expression level of *APE1* mRNA increased significantly after UUO, suggesting that it participates in the pathogenesis of tubulointerstitial fibrosis. In addition, immunohistochemical analysis showed that APE1 immunoreactivity was increased and predominantly localized to tubular epithelial cells after UUO, as shown in a previous report^9^, suggesting that APE1 in tubular epithelial cells exerts some effects on neighboring cells including tubular epithelial cells themselves and interstitial cells. It has been reported that APE1 localizes to essentially all cells in normal kidneys, which correlated with the function of APE1 in DNA repair. However, after UUO, the expression of APE1 was detected mainly in tubular epithelial cells and interstitial cells. These results suggest that APE1 has an inhibitory role in the development of tubulointerstitial fibrosis by acting on both tubular epithelial cells and interstitial cells.

To clarify the therapeutic effect of APE1, we injected a solution containing naked plasmid DNA encoding murine APE1 into the renal vein after UUO. Plasmid vectors have several advantages over viral vectors. A large quantity of highly purified plasmid DNA can be obtained easily and inexpensively without any apparent immune response mediated by the DNA vector^20^. Indeed, we demonstrated that this method can result in the transfer of naked plasmids. DNA including both pCAG-EGFP and pCAG-APE1 was subsequently observed in renal interstitial fibroblasts near the peritubular capillaries, as previously reported^14,15^. In this study, inflammation and fibrosis in the kidney after UUO were significantly reduced by APE1 transfection. Previous studies indicated that APE1 inhibits inflammatory pathways in murine macrophages by suppressing the expression of inducible NO synthase (iNOS or Nos2)^21^. Although a recent study reported that fibrosis could contribute to tissue repair and exert beneficial effects on renal injury, we confirmed that tubular injury was also significantly reduced by APE1 transfection, with a parallel marked decrease in tubular injury-associated gene transcripts such as *Lcn2 (Ngal)* and *Havcr1 (Kim1)*. Taken together, these results suggest that APE1-transfected fibroblasts play a suppressive role in the development of tubulointerstitial fibrosis, tubular injury, and inflammation *in vivo* partly via an autocrine/paracrine mechanism. In terms of results that fibrillar collagen-encoding gene transcripts were not significant differences in between pCAG-EGFP and pCAG-APE1-injected UUO kidneys, this might be due to the relatively small number of mice examined.

The accumulation of oxidative stress, as detected by 8-OHdG, and oxidative stress-induced apoptosis, as detected by *Casp3* gene transcription, was significantly reduced in UUO kidneys after APE1 transfection. The APE1 enzyme has recently been characterized as a multifunctional protein that responds to various oxidative stressors to protect the organ, which occurs via two intrinsic pathways as follows: (a) the base excision repair pathway that exhibits DNA repair activity, regulated by the C-terminal region, and (b) the redox system (Ref-1) that activates various transcription factors, regulated by the N-terminal region^4,5^. Bioinformatics analyses identified metabolic process and catalytic activity as the most abundant GO category for biological process and molecular function, respectively. Among metabolic process and catalytic activity terms, *Nos2*, *Ptgs2*, *Casp1*, *Casp4*, *Casp7*, *Casp12*, *Jak2*, and *Jak3* were significantly down-regulated by APE1 transfection. Caspases are cysteine proteases that play important roles in different processes including apoptosis and inflammation^22^. Caspase-1, caspase-4, and capase-12 are inflammatory caspases, whereas caspase-3 and caspase-7 comprise apoptotic executioners^22–24^. The Janus kinase (JAK) tyrosine kinase family and JAK/STAT signal transduction pathway might function during fibrogenesis in various organs including the kidney, heart, lung, liver, and bone marrow by promoting oxidative stress-induced apoptosis^25–27^. These findings suggest that APE1 suppresses oxidative stress-induced apoptosis by inhibiting the activation of multiple caspases and JAK/STAT signaling.

Current and previous strategies to design and develop therapeutics for kidney diseases favor reducing blood pressure and targeting overactivation of the immune response^1^. Macrophages in chronically-diseased tissue can drive disease progression by provoking local tissue injury and/or stimulating excessive fibrogenic responses^18^. In this study, bioinformatics analyses of whole tissue did not identify a specific receptor signaling pathway that was either activated or blocked by APE1. Rather, results suggested that APE1 has broad anti-inflammatory activity. Although most genes regulated by APE1 were found to be involved in enhancing metabolic functions and catalytic activity based on GO analyses of biological processes and molecular functions, we postulate that APE1 acts predominantly to inhibit inflammatory pathways based on results of the DAG and the fact that enhanced metabolic functions and catalytic activity are secondary consequences. We found that *Il6*, *Tnf*, *Ifng*, *Tgfb1*, *Nos2*, *Ptgs2*, and *Casp1*, as well as various chemokine families including MCP-1 and RANTES, were markedly down-regulated by APE1 transfection based on bioinformatics analyses. Inflammatory cytokines, chemokines, and chemokine receptors are well known to be involved in the progression of renal diseases including tubulointerstitial fibrosis^18,19^. Caspase-1 is also known to process the cytokines pro-IL-1β and pro-IL-18 into their active forms, thus triggering inflammation^23^. Taken together, these results suggest that APE1 has a potent inhibitory effect on the expression of these inflammatory cytokines and chemokines. However, we did not directly prove that APE1 decreases renal fibrosis by modulating the immune system. For example, APE1 was found to alter metabolic processes and catalytic activity, which might reduce renal fibrosis independently of the immune system. Further studies will be required to determine the direct role of APE1 in modulating the immune system and fibrosis in various models of chronic kidney disease.

In conclusion, APE1 transfection by retrograde renal vein injection is an effective method to counteract the progression of tubulointerstitial fibrosis in a mouse UUO model. APE1 has a protective effect on oxidative stress-induced apoptosis but also exerts anti-inflammatory effects by inhibiting many inflammatory cytokine and chemokine pathways in diseased kidneys. Our studies therefore identify APE1 transfection as a potential therapeutic strategy to control chronic fibrotic diseases of the kidney.

## Methods

### Ethics statement

All procedures including animal studies were conducted following the guidelines for the Care and Use of Laboratory Animals of the Ministry of Education, Culture, Sports, Science and Technology, Japan. The authors received approval from the Institutional Animal Care and Use Committees (IACUC)/ethics committee of Asahikawa Medical University institutional review board (protocol number 18083).

### Plasmid DNA

The mouse *APE1* gene with an N-terminal HA tag or EGFP was sub-cloned into the mammalian expression vector pCAG (Wako) using Ligation high (TOYOBO), creating pCAG-APE1 and pCAG-EGFP, respectively, according to the manufacturer’s instructions.

### Functional analysis of plasmid vectors

To evaluate APE1 and EGFP expression, pCAG-APE1 and pCAG-EGFP were transfected into BHK-21 cells using Polyethyleneimine Max (Polysciences) according to the manufacturer’s instructions. At 24 hours after transfection, cells were washed twice in PBS before fixing with 2% paraformaldehyde for 10 minutes. For antigen detection by fluorescence, primary antibodies against the following proteins were used for immunolabeling: APE1 (1:200, Novus), HA tag (1:200, Abcam). Primary antibodies were detected with fluorescence-conjugated, affinity-purified secondary antibody labeling (1:1000, Thermo Fisher). Samples were co-labeled with DAPI and mounted with ProLong Diamond (Thermo Fisher). Fluorescence images were captured using a BZ-X710 microscope (Keyence) as described previously^7^.

### Animal study

Plasmid DNA (200 μg) was diluted in PBS (1 μg/μl). To induce the development of renal interstitial fibrosis, UUO was performed according to a previously reported method^28^. In brief, male CD-1 mice, aged 10–12 weeks, were anesthetized with isoflurane and placed in an abdominal position with body temperature control. The left ureter was exposed through a small suprapelvic incision and ligated at two sites within the middle portion with a 3/0 silk thread. After the induction of UUO, the plasmid DNA solution was injected via the left renal vein as previously described^14,15^. Briefly, the left renal vein and artery were clamped with a microclip (Roboz). Immediately after the occlusion of the vessels, the plasmid DNA solution was injected into the renal vein within a few seconds using a 1-ml-capacity lure lock syringe (OSAKA CHEMICAL) with a 26-gauge needle (Nipro).

### Histology staining and evaluation

Kidneys were resected after systemic perfusion with ice-cold PBS. Paraffin-embedded sections of kidneys fixed with neutral-buffered formalin were used for Sirius red staining. Interstitial fibrosis was quantified as Sirius red-positive areas as previously reported^28^. The extent of tubular injury in the renal cortex was graded as follows: 1, 0–25%; 2, 25–50%; 3, 50–75%; and 4, 75–100%, as previously reported^29^.

### Immunofluorescence staining and evaluation

Mouse tissues were prepared and stained as described previously^30^. Briefly, kidneys were fixed in PLP solution for 2 hours and washed in 18% sucrose solution overnight prior to cryopreservation and cryosectioning (to a thickness of 7 μm). For antigen detection by fluorescence, primary antibodies against the following proteins were used for immunolabeling: GFP-FITC (1:200, Abcam), α-SMA-Cy3 (1:200, clone 1A4, Sigma-Aldrich), F4/80 (1:200, Thermo Fisher), and 8-OHdG (1:50, Japan Institute for the Control of Aging). Primary antibodies were detected with fluorescence-conjugated, affinity-purified secondary antibody labeling (1:1000, Thermo Fisher). Samples were co-labeled with DAPI and mounted with ProLong Diamond (Thermo Fisher). Fluorescence images were captured using a BZ-X710 microscope (Keyence).

### Quantitative RT-PCR

Total RNA was extracted using TRIzol (Thermo Fisher) and an RNeasy Mini Kit (QIAGEN) according to manufacturer’s instructions. Purity was determined based on A_260_ to A_280_ ratios. cDNA was synthesized using oligo(dT) and random primers (iScript, Bio-Rad). Quantitative RT-PCR (qPCR) was performed using TaqMan Gene Expression Master Mix (Thermo Fisher) on a LightCycler 96 (Roche). The specific TaqMan probe sets used for qPCR are listed in Supplementary Table 3. All values were normalized to *Gapdh* and graphed as fold-change versus control.

### RNA-seq analysis

RNA sequencing was performed by Novogene Bioinformatics Technology (Beijing, China) as described previously^31^. PCA, a method to produce low-dimensional projections of high-dimensional data, was performed based on gene expression variability across all 12 samples from the animal experiments (pCAG-EGFP sham, pCAG-APE1 sham, pCAG-EGFP UUO, pCAG-APE1 UUO). Downstream analysis was performed using a combination of programs including Bowtie2^32^, Tophat2^33^, HTseq^34^, and Cufflink^35^. Alignments were parsed using the Tophat program and differential expression was determined through DESeq2/DEGseq^36^. GO and KEGG enrichment were implemented by the GOseq R package^37^ and KOBAS^38^. Gene fusion and differences in alternative splicing events were detected by MISO and TopHatfusion software^39^. GO terms with a corrected P-value less than 0.05 were considered significantly enriched, based on differentially expressed genes. KEGG is a database resource for understanding high-level functions and utilities of the biological system such as the cell, organism, and ecosystem based on molecular-level information, especially large-scale molecular datasets generated by genome sequencing and other high-throughput experimental technologies (http://www.genome.jp/kegg/). Detailed procedures for this analysis are included in the Supplementary Methods.

### Statistics

Data are presented as the mean ± SEM, and differences among groups were assessed by performing an ANOVA with Tukey’s post hoc analysis. P values less than 0.05 were considered significant.

## Supplementary information

Supplemental methods, figure and tables

## Data Availability

The authors confirm that the data supporting the findings of this study are available within the article and its Supplementary Materials.

## References

[1] Romagnani P (2017). Chronic kidney disease. Nat Rev Dis Primers.

[2] Maruyama K (2016). Malnutrition, renal dysfunction and left ventricular hypertrophy synergistically increase the long-term incidence of cardiovascular events. Hypertens. Res..

[3] Humphreys BD (2018). Mechanisms of Renal Fibrosis. Annu. Rev. Physiol..

[4] Kelley MR, Georgiadis MM, Fishel ML (2012). APE1/Ref-1 role in redox signaling: translational applications of targeting the redox function of the DNA repair/redox protein APE1/Ref-1. Curr Mol Pharmacol.

[5] Thakur S (2014). APE1/Ref-1 as an emerging therapeutic target for various human diseases: phytochemical modulation of its functions. Exp. Mol. Med..

[6] Yamauchi A (2013). Apurinic/apyrimidinic endonucelase 1 maintains adhesion of endothelial progenitor cells and reduces neointima formation. Am J Physiol Heart Circ Physiol.

[7] Aonuma T (2016). Apoptosis-Resistant Cardiac Progenitor Cells Modified With Apurinic/Apyrimidinic Endonuclease/Redox Factor 1 Gene Overexpression Regulate Cardiac Repair After Myocardial Infarction. Stem Cells Transl Med.

[8] Kim JN (2012). The role of apurinic/apyrimidinic endonuclease on the progression of streptozotocin-induced diabetic nephropathy in rats. Acta Histochem..

[9] Aamann MD (2016). Unilateral ureteral obstruction induces DNA repair by APE1. Am J Physiol Renal Physiol.

[10] Chang IY (2015). Apurinic/apyrimidinic endonuclease 1 on aging-associated deteriorations in rat kidneys. Free Radic. Res..

[11] Kanehisa M, Goto S (2000). KEGG: kyoto encyclopedia of genes and genomes. Nucleic Acids Res.

[12] Kanehisa M (2017). KEGG: new perspectives on genomes, pathways, diseases and drugs. Nucleic Acids Res.

[13] Kanehisa M (2019). New approach for understanding genome variations in KEGG. Nucleic Acids Res.

[14] Kameda S (2004). Kidney-targeted naked DNA transfer by retrograde injection into the renal vein in mice. Biochem. Biophys. Res. Commun..

[15] Fujii N (2006). Targeting of interstitial cells using a simple gene-transfer strategy. Nephrol. Dial. Transplant..

[16] Rossi M (2017). Specific expression of heme oxygenase-1 by myeloid cells modulates renal ischemia-reperfusion injury. Sci Rep.

[17] Duffield JS (2014). Cellular and molecular mechanisms in kidney fibrosis. J. Clin. Invest..

[18] Eming SA, Wynn TA, Martin P (2017). Inflammation and metabolism in tissue repair and regeneration. Science.

[19] Anders HJ, Vielhauer V, Schlondorff D (2003). Chemokines and chemokine receptors are involved in the resolution or progression of renal disease. Kidney Int..

[20] Taniyama Y (2012). Therapeutic option of plasmid-DNA based gene transfer. Curr Top Med Chem.

[21] Song JD (2008). Redox factor-1 mediates NF-kappaB nuclear translocation for LPS-induced iNOS expression in murine macrophage cell line RAW 264.7. Immunology.

[22] Parrish, A. B., Freel, C. D. & Kornbluth, S. Cellular mechanisms controlling caspase activation and function. *Cold Spring Harb Perspect Biol* **5** (2013). 10.1101/cshperspect.a008672 PMC3660825 23732469

[23] Sollberger G (2014). Caspase-1: the inflammasome and beyond. Innate Immun.

[24] Garcia de la Cadena S, Massieu L (2016). Caspases and their role in inflammation and ischemic neuronal death. Focus on caspase-12. Apoptosis.

[25] Harrison C (2012). JAK inhibition with ruxolitinib versus best available therapy for myelofibrosis. N. Engl. J. Med..

[26] Koike K (2014). Protective role of JAK/STAT signaling against renal fibrosis in mice with unilateral ureteral obstruction. Clin. Immunol..

[27] Ibrahim MK (2017). Increased incidence of cytomegalovirus coinfection in HCV-infected patients with late liver fibrosis is associated with dysregulation of JAK-STAT pathway. Sci Rep.

[28] Nakagawa N (2012). The intrinsic prostaglandin E2-EP4 system of the renal tubular epithelium limits the development of tubulointerstitial fibrosis in mice. Kidney Int..

[29] Nakamura J (2019). Myofibroblasts acquire retinoic acid-producing ability during fibroblast-to-myofibroblast transition following kidney injury. Kidney Int..

[30] Nakagawa N (2016). Pentraxin-2 suppresses c-Jun/AP-1 signaling to inhibit progressive fibrotic disease. JCI Insight.

[31] Tang Q (2017). Diplosporous development in Boehmeria tricuspis: Insights from de novo transcriptome assembly and comprehensive expression profiling. Sci Rep.

[32] Langmead B, Salzberg SL (2012). Fast gapped-read alignment with Bowtie 2. Nat Methods.

[33] Kim D (2013). TopHat2: accurate alignment of transcriptomes in the presence of insertions, deletions and gene fusions. Genome Biol.

[34] Anders S, Pyl PT, Huber W (2015). HTSeq–a Python framework to work with high-throughput sequencing data. Bioinformatics.

[35] Trapnell C (2013). Differential analysis of gene regulation at transcript resolution with RNA-seq. Nat. Biotechnol..

[36] Wang L, Feng Z, Wang X, Zhang X (2010). DEGseq: an R package for identifying differentially expressed genes from RNA-seq data. Bioinformatics.

[37] Young MD, Wakefield MJ, Smyth GK, Oshlack A (2010). Gene ontology analysis for RNA-seq: accounting for selection bias. Genome Biol.

[38] Mao X, Cai T, Olyarchuk JG, Wei L (2005). Automated genome annotation and pathway identification using the KEGG Orthology (KO) as a controlled vocabulary. Bioinformatics.

[39] Kim D, Salzberg SL (2011). TopHat-Fusion: an algorithm for discovery of novel fusion transcripts. Genome Biol.

