# Supplementary material for: The antioxidant and DNA-repair enzyme apurinic/apyrimidinic endonuclease 1 limits the development of tubulointerstitial fibrosis partly by modulating the immune system
Source: Sci Rep. 2019 May 24;9:7823. doi: 10.1038/s41598-019-44241-z (PMC6534557; doi:10.1038/s41598-019-44241-z)
Supplement: Supplementary file 1 — Supplemental methods, figure and tables [file 41598_2019_44241_MOESM1_ESM.pdf]

The antioxidant and DNA-repair enzyme apurinic/apyrimidinic endonuclease 1 limits the development of tubulointerstitial fibrosis partly by modulating the immune system

Keisuke Maruyama[1], Naoki Nakagawa[1], Tatsuya Aonuma[1], Yukihiro Saito[2], Taiki Hayasaka[1], Kohei Kano[1], Kiwamu Horiuchi[1], Naofumi Takehara[1,2], Jun-ichi Kawabe[3], Naoyuki Hasebe[1]

[1]Division of Cardiology, Nephrology, Respiratory and Neurology, Department of Internal Medicine, Asahikawa Medical University, Asahikawa, Japan
[2]Center for Advanced Research and Education, Asahikawa Medical University, Asahikawa, Japan
[3]Department of Cardiovascular Regeneration and Innovation, Asahikawa Medical University, Japan

Supplementary Method

Supplementary Figure 1. Principal component analysis (PCA) comparing mice subjected to unilateral obstructed ureter (UUO) procedures and sham-operated mice, injected with pCAG-APE1 or pCAG-EGFP (control) vectors

Supplementary Table 1. Significant differentially expression genes of catalytic activity between pCAG-EGFP (control) and pCAG-APE1 injected kidneys after UUO

Supplementary Table 2. Significant differentially expression genes of metabolic process between pCAG-EGFP (control) and pCAG-APE1 injected kidneys after UUO

Supplementary Table 3. qPCR primers list

Method

Data Analysis

Downstream analysis was performed using a combination of programs including Bowtie2, Tophat2, HTseq, Cufflink and our wrapped scripts. Alignments were parsed using Tophat program and differential expressions were determined through DESeq2/DEGseq. GO and KEGG enrichment were implemented by the GOseq R package and KOBAS. Gene fusion and difference of alternative splicing event were detected by MISO and TopHatfusion software

Reads mapping to the reference genome

Reference genome and gene model annotation files were downloaded from genome website browser (NCBI/UCSC/Ensembl) directly. Indexes of the reference genome were built using Bowtie v2.0.6 and paired-end clean reads were aligned to the reference genome using TopHat v2.0.9. Bowtie uses a BWT(Burrows-Wheeler Transformer) algorithm for mapping reads to the genome and Tophat can generate a database of splice junctions based on the gene model annotation file and thus achieve a better mapping result than other non-splice mapping tools.

Quantification of gene expression level

HTSeq v0.6.1 was used to count the read numbers mapped of each gene. And then RPKM of each gene was calculated based on the length of the gene and reads count mapped to this gene. RPKM, Reads Per Kilobase of exon model per Million mapped reads, considers the effect of sequencing depth and gene length for the reads count at the same time, and is currently the most commonly used method for estimating gene expression levels (Mortazavi et al., 2008).

Differential expression analysis

(For DESeq2 with biological replicates) Differential expression analysis between two conditions/groups (two biological replicates per condition) was performed using the DESeq2 R package (2_1.6.3). DESeq2 provide statistical routines for determining differential expression in digital gene expression data using a model based on the negative binomial distribution. The resulting P-values were adjusted using the Benjamini and Hochberg's approach for controlling the False Discovery Rate(FDR). Genes with an adjusted P-value <0.05 found by DESeq2 were assigned as differentially expressed.

(For DEGSeq without biological replicates) Prior to differential gene expression analysis, for each sequenced library, the read counts were adjusted by edgeR program package through one scaling normalized factor. Differential expression analysis of two conditions was performed using the DEGSeq R package (1.12.0). The P values were adjusted using the Benjamini & Hochberg method. Corrected P-value of 0.005 and log2(Fold change) of 1 were set as the threshold for significantly differential expression.

The Venn diagrams were prepared using the function vennDiagram in R based on the gene list for different group.

Correlations

To allow for log adjustment, genes with 0 RPKM are assigned a value of 0.001. Correlation

were determined using the cor.test function in R with options set alternative =”greater” and method = “Spearman”.

Clustering

To identify the correlation between difference, we clustered different samples using expression level RPKM to see the correlation using hierarchical clustering distance method with the function of heatmap, SOM(Self-organization mapping) and kmeans using silhouette coefficient to adapt the optimal classification with default parameter in R.

GO and KEGG enrichment analysis of differentially expressed genes

Gene Ontology (GO) enrichment analysis of differentially expressed genes was implemented by the GOseq R package, in which gene length bias was corrected. GO terms with corrected P-value less than 0.05 were considered significantly enriched by differential expressed genes.

KEGG is a database resource for understanding high-level functions and utilities of the biological system, such as the cell, the organism and the ecosystem, from molecular-level information, especially large-scale molecular datasets generated by genome sequencing and other high-through put experimental technologies (http://www.genome.jp/kegg/). We used KOBAS software to test the statistical enrichment of differential expression genes in KEGG pathways.

PPI analysis of differentially expressed genes

PPI analysis of differentially expressed genes was based on the STRING database, which contained known and predicted Protein-Protein Interactions. For the species existing in the database(like human and mouse), we constructed the networks by extracting the target gene lists from the database.

Novel transcripts prediction and annotation

The Cufflinks v2.1.1 was used to assemble the scaffold , construct and identify both known and novel transcripts from TopHat alignment results. And Hmmscan was used to annotated the probable function of the novel transcripts against Pfam database.

Fusion gene analysis

For human and mouse data, we use SOAPfuse and Tophat fusion to identify fusion genes in tumor samples, then visualize results.

SOAPfuse identify fusion transcripts from paired-end RNA-Seq data. SOAPfuse applies an improved partial exhaustion algorithm to construct a library of fusion junction sequences, which can be used to efficiently identify fusion events, and employs a series of filters to nominate high-confidence fusion transcripts. Compared with other released tools, SOAPfuse achieves higher detection efficiency and consumed less computing resources. It also can predict fusion point and visualize results.

TopHatfusion was applied for gene fusion discovery using RNA-Seq data. The software invoked bowtie to align reads to spliced genes, genome, and UniGene sequences in paired end mode. It used clusters of discordant paired end alignments to inform a split read alignment analysis for finding fusion boundaries. The software also employed a number of heuristic filters in an attempt to reduce the number of false positives and produced a fully annotated output for each predicted fusion.

SNP analysis

We deal with the bam alignment results of each sample by using picard tools(v1.111) and samtools(v0.1.18), including reorder, sort, add head information, mark duplicates, local realignment around indels and base qulaity score recalibration. Then we call snp by the tool HaplotypeCaller in GATK3.4 version. Finally, we ues annovar to do SNP annotation against dbSNP database and some other database.

Alternative splicing analysis

MISO v0.5.2 was used for alternative splicing event analysis, difference detection and visualization. MISO (Mixture-of-Isoforms) is a probabilistic framework that quantitates the expression level of alternatively spliced genes from RNA-Seq data, and identifies differentially regulated isoforms or exons across samples. By modeling the generative process by which reads are produced from isoforms in RNA-Seq, the MISO model uses Bayesian inference to compute the probability that a read originated from a particular isoform.MISO treats the expression level of a set of isoforms as a random variable and estimates a distribution over the values of this variable. The estimation algorithm is based on sampling, and falls in the family of techniques known as Markov Chain Monte Carlo ("MCMC"). PSI(Percent Spliced In) was used in the difference detection with $\Delta\psi$ bigger than 0.2 and bayes factor >10.

Differentially expressed gene annotation

TFCat and Cosmic database were used to annotate the differential expressed gene. TFCat is a curated catalog of mouse and human transcription factors (TF) based on a reliable core collection of annotations obtained by expert review of the scientific literature. COSMIC is a database designed to store and display somatic mutation information and related details which contains information relating to human cancers.

Data access

The high-throughput sequencing data from this study have been submitted to the NCBI Sequence Read Archive (SRA) under accession number ####

Acknowledgements

This work was supported by grant from ###. We thank ###### for technical supports and deep discussion.

References

Anders, S.(2010). HTSeq: Analysing high-throughput sequencing data with Python.(HTSeq)

Anders, S., and Huber, W. (2010). Differential expression analysis for sequence count data. Genome Biol.(DESeq)

Anders, S. and Huber, W. (2012). Differential expression of RNA-Seq data at the gene level-the DESeq package.(DESeq)

Kanehisa, M., M. Araki, et al. (2008). KEGG for linking genomes to life and the environment. Nucleic acids research.(KEGG)

Langmead, B., Trapnell, C., Pop, M. & Salzberg, S.L. (2009). Ultrafast and memory-efficient alignment of short DNA sequences to the human genome. Genome Biol.(Bowtie)

Langmead, B. and S. L. Salzberg (2012). Fast gapped-read alignment with Bowtie 2. Nature methods.(Bowtie 2)

Mao, X., Cai, T., Olyarchuk, J.G., Wei, L. (2005). Automated genome annotation and pathway identification using the KEGG Orthology (KO) as a controlled vocabulary. Bioinformatics.(KOBAS)

Marioni, J. C., C. E. Mason, et al. (2008). RNA-seq: an assessment of technical reproducibility

and comparison with gene expression arrays. Genome research.
Altshuler, D, Gabriel, S, Daly, M, DePristo, MA. 2010. The Genome Analysis Toolkit: a MapReduce framework for analyzing next-generation DNA sequencing data. Genome Research.(GATK)
Mortazavi, A., B. A. Williams, et al. (2008). Mapping and quantifying mammalian transcriptomes by RNA-Seq. Nature methods.
Robinson, M. D., McCarthy, D. J. & Smyth, G. K. edgeR: a Bioconductor package for differential expression analysis of digital gene expression data. Bioinformatics.(edgeR)
Shannon et al. (2003).Cytoscape: a software environment for integrated models of biomolecular interaction networks. (Cytoscape)
Trapnell, C. et al. (2010).Transcript assembly and quantification by RNA-seq reveals unannotated transcripts and isoform switching during cell differentiation. Nat. Biotechnol.(Cufflinks)
Trapnell, C., Pachter, L., and Salzberg, S.L. (2009). TopHat: discovering splice junctions with RNA-Seq. Bioinformatics.(TopHat)
Trapnell, C., A. Roberts, et al. (2012). Differential gene and transcriptexpression analysis of RNA-seq experiments with TopHat and Cufflinks. nature protocols.(Tophat & Cufflinks)
Wang, L.Feng, Z.Wang, X.Zhang, X. (2010). DEGseq: an R package for identifying differentially expressed genes from RNA-seq data. Bioinformatics.(DEGseq)
Wang, Z., M. Gerstein, et al. (2009). RNA-Seq: a revolutionary tool for transcriptomics. Nature Reviews Genetics.
Young, M. D., Wakefield, M. J., Smyth, G. K., and Oshlack, A. (2010).Gene ontology analysis for RNA-seq: accounting for selection bias. Genome Biology.(GOseq)
Fulton DL1, Sundararajan S, Badis G, et al. (2009). TFCat: the curated catalog of mouse and human transcription factors. Genome Biol. 10(3):R29.(TFCat)
Chepelev I, Wei G, Tang Q, et al. (2009). Detection of single nucleotide variations in expressed exons of the human genome using RNA-Seq. Nucleic acids research 37, e106-e106. (SNP)
Cingolani, P., et. al. (2012). Using Drosophila melanogaster as a model for genotoxic chemical mutational studies with a new program, SnpSift. Frontiers in Genetics 3.(SnpSift)
Yarden Katz, Eric T. Wang, Edoardo M, et al. (2010). Analysis and design of RNA sequencing experiments for identifying isoform regulation. Nature Methods 7, 1009-1015 (MISO)

Figure S1

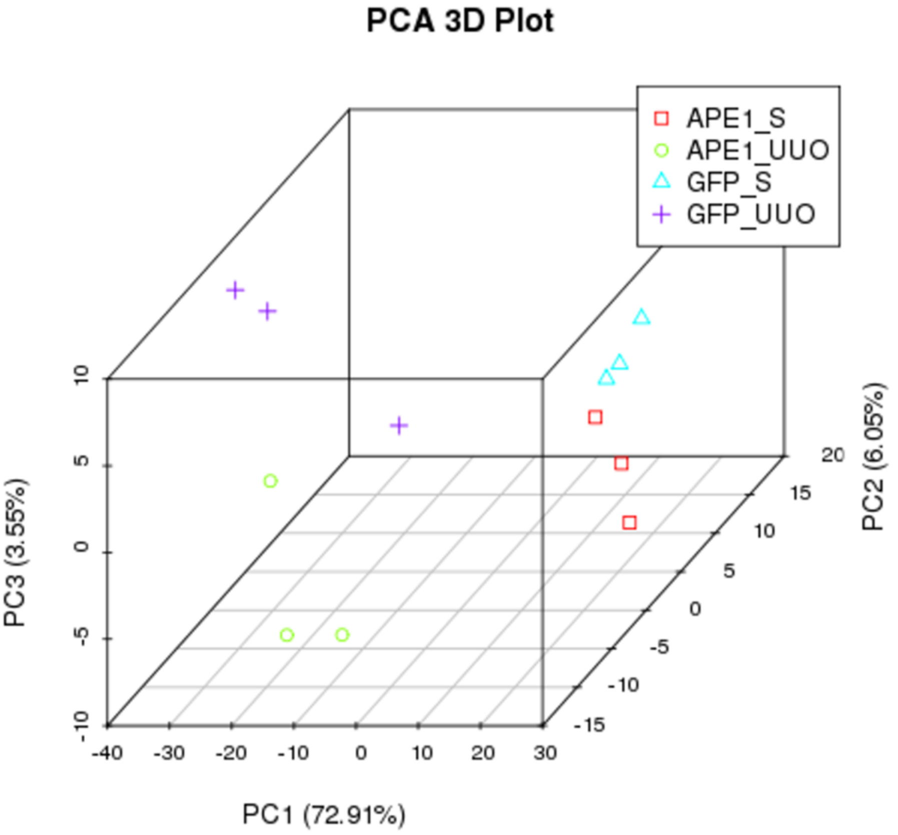

Maruyama K, et al.

Supplementary Table 1. Significant differentially expression genes of catalytic activity between pCAG-EGFP (control) and pCAG-APE1 injected kidneys after UUO

ID	Gene	EGFP_readcount	APE1_readcount	log2(fold change)	P value	Regulation
ENSMUSG00000048087	Gm4737	563.31	1734.26	1.62	1.22E-26	Up
ENSMUSG00000074639	BC089597	293.81	989.59	1.75	1.48E-09	Up
ENSMUSG00000078650	G6pc	543.06	2905.04	2.42	3.28E-09	Up
ENSMUSG00000040170	Fmo2	1626.91	3420.16	1.07	1.42E-08	Up
ENSMUSG00000010651	Acaa1b	821.47	3095.02	1.91	2.10E-08	Up
ENSMUSG00000020988	L2hgdh	1560.90	3078.10	0.98	2.36E-08	Up
ENSMUSG00000042102	Dmgdh	641.22	1433.22	1.16	5.08E-08	Up
ENSMUSG00000066097	Cyp2j11	897.69	2150.63	1.26	6.83E-08	Up
ENSMUSG00000040740	Slc25a34	298.64	646.45	1.11	1.87E-07	Up
ENSMUSG00000021416	Eci3	524.56	1232.06	1.23	1.39E-06	Up
ENSMUSG00000020051	Pah	2515.14	6869.66	1.45	2.08E-06	Up
ENSMUSG00000040860	Crocc	439.35	922.52	1.07	2.13E-06	Up
ENSMUSG00000023044	Csad	2389.49	5670.93	1.25	2.75E-06	Up
ENSMUSG00000028093	Acp6	870.41	1378.70	0.66	3.01E-06	Up
ENSMUSG00000033634	Cml2	699.31	1934.50	1.47	3.64E-06	Up
ENSMUSG00000001467	Cyp51	2322.59	5128.56	1.14	4.77E-06	Up
ENSMUSG00000026348	Acmsd	483.48	1941.67	2.01	6.55E-06	Up
ENSMUSG00000027070	Lrp2	15218.97	35951.69	1.24	1.80E-05	Up
ENSMUSG00000051262	Cml3	39.89	136.69	1.78	1.94E-05	Up
ENSMUSG00000045316	Fahd1	1137.50	2095.48	0.88	2.96E-05	Up
ENSMUSG00000042251	Pm20d1	879.83	2537.17	1.53	3.21E-05	Up

ENSMUSG00000000673	Haao	730.58	2163.22	1.57	3.34E-05	Up
ENSMUSG00000028179	Cth	1499.77	2854.48	0.93	3.85E-05	Up
ENSMUSG00000061959	Ces1e	61.17	242.24	1.99	3.85E-05	Up
ENSMUSG00000022821	Hgd	1776.90	3791.33	1.09	4.71E-05	Up
ENSMUSG00000066072	Cyp4a10	718.32	2669.18	1.89	4.72E-05	Up
ENSMUSG00000025731	0610011F06Rik	1665.85	2841.50	0.77	5.46E-05	Up
ENSMUSG00000062410	Hsd3b3	236.10	557.28	1.24	6.00E-05	Up
ENSMUSG00000029695	Aass	2851.23	5885.26	1.05	6.20E-05	Up
ENSMUSG00000027187	Cat	5367.64	10842.39	1.01	7.12E-05	Up
ENSMUSG00000079261	Gm15217	382.11	804.66	1.07	7.68E-05	Up
ENSMUSG00000035878	Hykk	2153.48	5025.78	1.22	8.53E-05	Up
ENSMUSG00000024781	Lipa	3209.62	5094.64	0.67	0.00012	Up
ENSMUSG00000003746	Man1a	3275.53	4883.07	0.58	0.00014	Up
ENSMUSG00000055373	Fut9	2945.78	6125.64	1.06	0.00015	Up
ENSMUSG00000022235	Cmbl	639.13	1591.64	1.32	0.00015	Up
ENSMUSG00000030004	Nat8	1131.80	3011.97	1.41	0.00016	Up
ENSMUSG00000046598	Bdh1	1963.73	3991.21	1.02	0.00017	Up
ENSMUSG00000027513	Pck1	4517.37	15664.93	1.79	0.00019	Up
ENSMUSG00000079495	Gm11128	262.92	1087.41	2.05	0.00019	Up
ENSMUSG00000024899	Papss2	1864.72	3403.00	0.87	0.00019	Up
ENSMUSG00000042073	Abhd14b	955.38	1795.67	0.91	0.00021	Up
ENSMUSG00000054619	Mettl7a1	3014.48	5945.04	0.98	0.00021	Up
ENSMUSG00000029482	Aacs	1666.42	3072.08	0.88	0.00027	Up
ENSMUSG00000028712	Cyp4a31	1481.13	2967.17	1.00	0.0003	Up

ENSMUSG00000069805	Fbp1	4459.42	11235.58	1.33	0.00031	Up
ENSMUSG00000079494	Cml5	139.16	493.27	1.83	0.00032	Up
ENSMUSG00000049624	Slc17a5	1954.50	2609.57	0.42	0.00032	Up
ENSMUSG00000058997	Vwa8	2657.09	4544.21	0.77	0.00033	Up
ENSMUSG00000041052	Slc7a13	5014.82	15201.38	1.60	0.00034	Up
ENSMUSG00000038286	Bphl	1944.94	3714.24	0.93	0.00038	Up
ENSMUSG00000080115	Mettl21b	56.74	124.46	1.13	0.00038	Up
ENSMUSG00000028527	Ak4	1936.75	6557.68	1.76	0.0004	Up
ENSMUSG00000003809	Gcdh	2349.78	4852.63	1.05	0.00043	Up
ENSMUSG00000037440	Vnn1	654.08	1488.26	1.19	0.00045	Up
ENSMUSG00000031445	Proz	40.60	136.50	1.75	0.0005	Up
ENSMUSG00000040181	Fmo1	3193.36	5983.43	0.91	0.0005	Up
ENSMUSG00000074254	Cyp2a4	2360.32	4804.48	1.03	0.0005	Up
ENSMUSG00000026730	Pter	6305.31	9976.00	0.66	0.00051	Up
ENSMUSG00000003849	Nqo1	275.36	583.55	1.08	0.00054	Up
ENSMUSG00000036395	Glb1l2	1694.72	2699.58	0.67	0.00054	Up
ENSMUSG00000024713	Pcsk5	613.63	855.93	0.48	0.00054	Up
ENSMUSG00000002769	Gnmt	114.10	277.79	1.28	0.00054	Up
ENSMUSG00000050097	Ces2b	83.78	270.29	1.69	0.00059	Up
ENSMUSG00000020182	Ddc	422.50	826.35	0.97	0.00062	Up
ENSMUSG00000024131	Slc3a1	5282.51	8056.38	0.61	0.00065	Up
ENSMUSG00000041426	Hibch	638.73	981.78	0.62	0.00066	Up
ENSMUSG00000041757	Plekha6	2146.22	3048.70	0.51	0.00068	Up
ENSMUSG00000060224	Pyroxd2	901.59	1561.33	0.79	0.0007	Up

ENSMUSG00000023830	Igf2r	2662.31	4055.62	0.61	0.00074	Up
ENSMUSG00000028756	Pink1	3329.04	5251.32	0.66	0.00078	Up
ENSMUSG00000035637	Grhpr	1841.53	3978.72	1.11	0.00082	Up
ENSMUSG00000001755	Coasy	2505.36	4743.61	0.92	0.00088	Up
ENSMUSG00000028607	Cpt2	1490.01	2683.21	0.85	0.00095	Up
ENSMUSG00000027610	Gss	3422.65	6133.76	0.84	0.00099	Up
ENSMUSG00000027761	Aadac	757.94	1460.50	0.95	0.00099	Up
ENSMUSG00000089694	Gm4477	25.45	114.29	2.17	0.00103	Up
ENSMUSG00000026200	Glb1l	1364.65	1973.14	0.53	0.00105	Up
ENSMUSG00000024978	Gpam	735.24	1515.33	1.04	0.0011	Up
ENSMUSG00000024747	Aldh1a7	172.76	448.39	1.38	0.0011	Up
ENSMUSG00000030972	Acsm5	532.07	1169.12	1.14	0.0012	Up
ENSMUSG00000022742	Cpox	1837.99	3318.77	0.85	0.00124	Up
ENSMUSG00000068086	Cyp2d9	717.61	1330.93	0.89	0.00127	Up
ENSMUSG00000003477	Inmt	1921.73	4636.02	1.27	0.0013	Up
ENSMUSG00000029378	Areg	51.05	158.21	1.63	0.0014	Up
ENSMUSG00000039450	Dcxr	801.57	1686.98	1.07	0.00142	Up
ENSMUSG00000025194	Abcc2	1662.16	4228.91	1.35	0.00147	Up
ENSMUSG00000029098	Acox3	1616.81	2772.56	0.78	0.00152	Up
ENSMUSG00000075304	Sp5	122.54	247.61	1.01	0.00159	Up
ENSMUSG00000015568	Lpl	3267.03	6418.91	0.97	0.00171	Up
ENSMUSG00000025911	Adhfe1	660.16	1777.23	1.43	0.00172	Up
ENSMUSG00000042797	Aqp11	123.69	306.74	1.31	0.00183	Up
ENSMUSG00000042410	Agps	5282.40	9585.08	0.86	0.00192	Up

ENSMUSG00000028518	Prkaa2	2086.92	3354.88	0.68	0.00198	Up
ENSMUSG00000057880	Abat	2524.35	4416.10	0.81	0.00204	Up
ENSMUSG00000049152	Ugt3a2	7745.11	16647.14	1.10	0.00215	Up
ENSMUSG00000027984	Hadh	4254.93	7334.95	0.79	0.00224	Up
ENSMUSG00000030283	St8sia1	1565.00	2750.15	0.81	0.00226	Up
ENSMUSG00000063730	Hsd3b2	1945.76	5020.18	1.37	0.00252	Up
ENSMUSG00000030935	Acsm3	6417.75	14595.21	1.19	0.00254	Up
ENSMUSG00000027227	Sord	5260.85	13447.71	1.35	0.00275	Up
ENSMUSG00000016756	Cmah	688.04	1168.00	0.76	0.00275	Up
ENSMUSG00000030747	Dgat2	1111.59	2338.12	1.07	0.00278	Up
ENSMUSG00000028571	Cyp2j13	3719.90	7865.39	1.08	0.00282	Up
ENSMUSG00000023019	Gpd1	1950.46	4503.32	1.21	0.00287	Up
ENSMUSG00000024892	Pcx	3379.63	7472.55	1.14	0.00287	Up
ENSMUSG00000031886	Ces2e	103.16	228.98	1.15	0.0029	Up
ENSMUSG00000019989	Enpp3	1147.16	1801.11	0.65	0.00291	Up
ENSMUSG00000035769	Xylb	3313.45	6406.80	0.95	0.00294	Up
ENSMUSG00000033533	Acsm1	1009.93	2701.01	1.42	0.00297	Up
ENSMUSG00000024158	Hagh	2226.67	3561.03	0.68	0.00324	Up
ENSMUSG00000030945	Acsm2	30871.05	74598.18	1.27	0.00333	Up
ENSMUSG00000019718	L3hypdh	451.99	837.96	0.89	0.00379	Up
ENSMUSG00000025937	Lactb2	5702.42	12499.12	1.13	0.00384	Up
ENSMUSG00000029802	Abcg2	4090.07	6408.96	0.65	0.00397	Up
ENSMUSG00000038704	Aspdh	221.53	770.01	1.80	0.00397	Up
ENSMUSG00000023262	Acy1	1979.75	3713.84	0.91	0.00408	Up

ENSMUSG00000020072	Pbld1	1030.15	2351.37	1.19	0.00432	Up
ENSMUSG00000024900	Cpt1a	5017.74	7439.15	0.57	0.0044	Up
ENSMUSG00000026687	Aldh9a1	2931.43	5700.28	0.96	0.0044	Up
ENSMUSG00000086784	Isoc2a	855.56	1374.70	0.68	0.00443	Up
ENSMUSG00000022210	Dhrs4	1897.78	3991.70	1.07	0.00455	Up
ENSMUSG00000025403	Shmt2	1676.77	3412.41	1.03	0.00455	Up
ENSMUSG00000075706	Gpx4	11153.01	15953.82	0.52	0.0048	Up
ENSMUSG00000022751	Nit2	1086.15	1697.94	0.64	0.00493	Up
ENSMUSG00000027371	Fahd2a	493.42	896.16	0.86	0.00493	Up
ENSMUSG00000037762	Slc16a9	640.99	1335.36	1.06	0.00495	Up
ENSMUSG00000022853	Ehhadh	5311.53	11921.88	1.17	0.00502	Up
ENSMUSG00000060376	Bckdha	2748.01	4453.71	0.70	0.00507	Up
ENSMUSG00000068587	Mgam	811.64	1321.64	0.70	0.00519	Up
ENSMUSG00000015337	Endog	216.81	347.68	0.68	0.0052	Up
ENSMUSG00000027999	Pla2g12a	241.37	400.14	0.73	0.00523	Up
ENSMUSG00000025158	Rfng	289.77	441.74	0.61	0.00526	Up
ENSMUSG00000078695	Cisd3	520.97	856.31	0.72	0.00526	Up
ENSMUSG00000003526	Prodh	1472.38	2456.30	0.74	0.00526	Up
ENSMUSG00000017718	Afmid	97.44	216.84	1.15	0.00534	Up
ENSMUSG00000090124	Ugt1a7c	254.69	473.64	0.90	0.00535	Up
ENSMUSG00000062908	Acadm	9180.51	18162.82	0.98	0.00539	Up
ENSMUSG00000025175	Fn3k	77.89	184.26	1.24	0.00548	Up
ENSMUSG00000030880	Polr3e	1090.75	1618.49	0.57	0.0056	Up
ENSMUSG00000090150	Acad11	1011.81	1498.64	0.57	0.0056	Up

ENSMUSG00000028307	Aldob	29575.02	53864.16	0.86	0.00569	Up
ENSMUSG00000059406	Tmprss9	55.30	202.69	1.87	0.00578	Up
ENSMUSG00000032047	Acat1	6646.06	12940.63	0.96	0.0058	Up
ENSMUSG00000028603	Scp2	7605.59	12506.75	0.72	0.00598	Up
ENSMUSG00000005547	Cyp2a5	235.69	776.03	1.72	0.00607	Up
ENSMUSG00000025968	Ndufs1	2495.39	3587.65	0.52	0.00612	Up
ENSMUSG00000033863	Klf9	1881.09	3058.09	0.70	0.00659	Up
ENSMUSG00000020150	Gamt	153.55	294.80	0.94	0.00689	Up
ENSMUSG00000004610	Etfb	3259.01	5777.03	0.83	0.00722	Up
ENSMUSG00000027809	Etfdh	2094.48	3012.69	0.52	0.00726	Up
ENSMUSG00000002010	Idh3g	3170.76	4400.44	0.47	0.00728	Up
ENSMUSG00000024782	Ak3	2893.93	4065.14	0.49	0.0073	Up
ENSMUSG00000014361	Mertk	443.86	717.51	0.69	0.00734	Up
ENSMUSG00000034371	Dak	4484.52	7479.86	0.74	0.00737	Up
ENSMUSG00000024921	Smarca2	1855.28	3602.52	0.96	0.00748	Up
ENSMUSG00000021048	Mthfd1	1925.06	2850.89	0.57	0.00768	Up
ENSMUSG00000025453	Nnt	2591.73	3332.18	0.36	0.00853	Up
ENSMUSG00000022229	Atp12a	44.85	125.27	1.48	0.00855	Up
ENSMUSG00000006378	Gcat	496.13	715.58	0.53	0.00866	Up
ENSMUSG00000031969	Acad8	1030.65	1429.27	0.47	0.00889	Up
ENSMUSG00000033488	BC026585	2798.30	4989.53	0.83	0.00892	Up
ENSMUSG00000030800	Prss8	3676.36	6099.64	0.73	0.00921	Up
ENSMUSG00000048578	Mlec	17381.96	26394.25	0.60	0.00955	Up
ENSMUSG00000058488	Kl	4236.74	7801.72	0.88	0.00957	Up

ENSMUSG00000023068	Nus1	5686.40	9031.93	0.67	0.00973	Up
ENSMUSG00000049091	Sephs2	3681.95	5971.13	0.70	0.00987	Up
ENSMUSG00000021263	Degs2	468.35	1288.57	1.46	0.00994	Up
ENSMUSG00000021238	Aldh6a1	5785.77	10670.23	0.88	0.00997	Up
ENSMUSG00000015405	Ace2	2168.29	2998.89	0.47	0.01011	Up
ENSMUSG00000032418	Me1	6950.58	12139.00	0.80	0.01024	Up
ENSMUSG00000029735	Tpk1	1135.92	1925.21	0.76	0.01032	Up
ENSMUSG00000033453	Adamts15	266.10	535.14	1.01	0.01041	Up
ENSMUSG00000025903	Lypla1	3511.67	5780.32	0.72	0.01042	Up
ENSMUSG00000042647	Acad12	341.83	573.51	0.75	0.01048	Up
ENSMUSG00000000385	Tmprss2	2730.74	3595.64	0.40	0.01054	Up
ENSMUSG00000019762	Iyd	415.59	745.05	0.84	0.01076	Up
ENSMUSG00000020098	Pcbd1	1599.67	2392.97	0.58	0.01076	Up
ENSMUSG00000023921	Mut	2054.89	2920.16	0.51	0.0108	Up
ENSMUSG00000041650	Pcca	1644.35	2504.43	0.61	0.01088	Up
ENSMUSG00000026179	Pnkd	1568.61	2496.44	0.67	0.01112	Up
ENSMUSG00000020681	Ace	4729.51	8191.56	0.79	0.01122	Up
ENSMUSG00000044475	Ascc1	779.10	1268.51	0.70	0.01127	Up
ENSMUSG00000061906	Ugt2b38	4775.07	11172.86	1.23	0.01128	Up
ENSMUSG00000063683	Glyat	3673.57	5958.70	0.70	0.01129	Up
ENSMUSG00000025486	Sirt3	557.06	1032.55	0.89	0.01129	Up
ENSMUSG00000027668	Mfn1	1858.82	2365.92	0.35	0.01141	Up
ENSMUSG00000051166	Eml5	532.41	856.50	0.69	0.01141	Up
ENSMUSG00000027896	Slc16a4	658.77	1307.34	0.99	0.01144	Up

ENSMUSG00000026958	Dpp7	2087.22	2890.83	0.47	0.01146	Up
ENSMUSG00000028194	Ddah1	3761.11	5642.90	0.59	0.01158	Up
ENSMUSG00000028973	Abcb8	1065.16	1515.44	0.51	0.01185	Up
ENSMUSG00000025209	Peo1	566.02	789.24	0.48	0.01187	Up
ENSMUSG00000021033	Gstz1	1822.21	3193.35	0.81	0.0119	Up
ENSMUSG00000072664	Ugt3a1	2545.47	5406.20	1.09	0.0119	Up
ENSMUSG00000006345	Ggt1	13741.98	27668.06	1.01	0.01232	Up
ENSMUSG00000032607	Amt	752.81	1093.39	0.54	0.01267	Up
ENSMUSG00000032540	Abhd5	668.37	904.71	0.44	0.01316	Up
ENSMUSG00000025545	Clybl	715.81	1199.15	0.74	0.01328	Up
ENSMUSG00000076441	Ass1	7858.12	14642.73	0.90	0.01339	Up
ENSMUSG00000024313	Mep1b	1108.66	2313.44	1.06	0.01353	Up
ENSMUSG00000029053	Prkcz	1289.98	1945.87	0.59	0.01372	Up
ENSMUSG00000053897	Slc39a8	938.61	1578.54	0.75	0.01406	Up
ENSMUSG00000030541	Idh2	4091.58	5510.32	0.43	0.01418	Up
ENSMUSG00000006313	Upk1a	90.17	218.22	1.28	0.01439	Up
ENSMUSG00000008226	Scrn3	558.87	896.58	0.68	0.01451	Up
ENSMUSG00000038173	Enpp6	807.70	1423.72	0.82	0.01451	Up
ENSMUSG00000032527	Pccb	4342.55	5807.48	0.42	0.01469	Up
ENSMUSG00000030630	Fah	3363.43	5563.68	0.73	0.0147	Up
ENSMUSG00000042118	Bhmt2	629.58	1482.12	1.24	0.01519	Up
ENSMUSG00000023122	Sult1c2	2134.09	3788.19	0.83	0.01531	Up
ENSMUSG00000030522	Mtmr10	540.92	727.34	0.43	0.01531	Up
ENSMUSG00000012187	Mogat1	845.60	2049.27	1.28	0.01596	Up

ENSMUSG00000030102	Itpr1	5802.07	7595.91	0.39	0.01596	Up
ENSMUSG00000053644	Aldh7a1	3190.94	4994.45	0.65	0.01616	Up
ENSMUSG00000028088	Fmo5	633.72	1500.57	1.24	0.01683	Up
ENSMUSG00000024041	Cryaa	15.28	59.18	1.95	0.0173	Up
ENSMUSG00000027406	Idh3b	3610.63	4914.66	0.44	0.0173	Up
ENSMUSG00000032387	Rbpms2	1607.68	2387.94	0.57	0.01732	Up
ENSMUSG00000004789	Dlst	5257.12	7734.95	0.56	0.01748	Up
ENSMUSG00000015478	Rnf5	918.15	1325.02	0.53	0.01754	Up
ENSMUSG00000022445	Cyp2d26	75.64	165.54	1.13	0.01773	Up
ENSMUSG00000051169	Rpusd3	157.07	253.27	0.69	0.01773	Up
ENSMUSG00000015536	Mocs2	867.40	1381.71	0.67	0.01781	Up
ENSMUSG00000067916	Gm13139	1.52	36.48	4.59	0.01797	Up
ENSMUSG00000074064	Mlycd	821.03	1406.94	0.78	0.01797	Up
ENSMUSG00000093930	Hmgcs1	6247.96	9759.09	0.64	0.01818	Up
ENSMUSG00000024386	Proc	2153.82	4010.09	0.90	0.0186	Up
ENSMUSG00000020623	Map2k6	200.77	333.60	0.73	0.01862	Up
ENSMUSG00000041168	Lonp1	3362.54	4576.20	0.44	0.019	Up
ENSMUSG00000037916	Ndufv1	4375.55	6505.67	0.57	0.01904	Up
ENSMUSG00000016194	Hsd11b1	2840.86	5456.41	0.94	0.01952	Up
ENSMUSG00000029311	Hsd17b11	3248.70	6648.62	1.03	0.01953	Up
ENSMUSG00000037542	Aldh8a1	2740.57	5251.15	0.94	0.01959	Up
ENSMUSG00000043681	Fam25c	33.32	82.72	1.31	0.0197	Up
ENSMUSG00000061838	Suclg2	4849.28	6713.32	0.47	0.02007	Up
ENSMUSG00000022186	Oxct1	10738.21	15963.74	0.57	0.02017	Up

ENSMUSG00000084854	Gm12678	109.20	301.31	1.46	0.02048	Up
ENSMUSG00000025059	Gyk	5900.44	10765.90	0.87	0.02099	Up
ENSMUSG00000020334	Slc22a4	608.61	841.23	0.47	0.02121	Up
ENSMUSG00000090231	Cfb	288.72	457.96	0.67	0.02129	Up
ENSMUSG00000020774	Aspa	437.12	1035.25	1.24	0.0215	Up
ENSMUSG00000024869	Nudt8	411.56	660.93	0.68	0.02151	Up
ENSMUSG00000029455	Aldh2	9177.69	13133.74	0.52	0.0216	Up
ENSMUSG00000027332	Ivd	3460.35	5537.55	0.68	0.0217	Up
ENSMUSG00000084350	Znf41-ps	0.00	30.19	#DIV/0!	0.02177	Up
ENSMUSG00000052520	Cyp2j5	17214.83	25630.21	0.57	0.02211	Up
ENSMUSG00000029273	Sult1d1	4150.43	7242.98	0.80	0.02212	Up
ENSMUSG00000023092	Fhl1	2345.52	4157.07	0.83	0.02214	Up
ENSMUSG00000041372	B4galnt3	616.77	902.31	0.55	0.02229	Up
ENSMUSG00000032098	Treh	1495.27	2988.46	1.00	0.02249	Up
ENSMUSG00000050645	Defb19	5.82	21.46	1.88	0.02271	Up
ENSMUSG00000046352	Gjb2	887.32	1610.08	0.86	0.02316	Up
ENSMUSG00000021322	Aoah	655.27	1148.91	0.81	0.02317	Up
ENSMUSG00000021102	Glrx5	1947.07	2785.40	0.52	0.02337	Up
ENSMUSG00000027709	Mccc1	1815.48	3103.60	0.77	0.0234	Up
ENSMUSG00000020189	Osbpl8	3666.92	5333.92	0.54	0.02369	Up
ENSMUSG00000020664	Dld	4140.68	5492.60	0.41	0.02372	Up
ENSMUSG00000020163	Uqcr11	3033.99	4233.33	0.48	0.02383	Up
ENSMUSG00000040658	Dnph1	276.11	550.42	1.00	0.02399	Up
ENSMUSG00000060227	Casc4	907.60	1576.01	0.80	0.02402	Up

ENSMUSG00000090817	Gm4450	1401.79	2430.78	0.79	0.02404	Up
ENSMUSG00000024694	Keg1	11334.90	20859.47	0.88	0.02412	Up
ENSMUSG00000033610	Pank1	3457.62	6713.72	0.96	0.02416	Up
ENSMUSG00000038843	Gcnt1	6537.56	9026.78	0.47	0.02453	Up
ENSMUSG00000021947	Cryl1	1608.45	3015.96	0.91	0.02504	Up
ENSMUSG00000020321	Mdh1	10424.82	15034.10	0.53	0.02521	Up
ENSMUSG00000025465	Echs1	2541.71	3633.81	0.52	0.02614	Up
ENSMUSG00000024130	Abca3	12100.95	17607.50	0.54	0.02647	Up
ENSMUSG00000021707	Dhfr	601.43	954.29	0.67	0.02667	Up
ENSMUSG00000031844	Hsd17b2	363.77	583.88	0.68	0.02711	Up
ENSMUSG00000014905	Dnajb9	1418.90	1807.31	0.35	0.02714	Up
ENSMUSG00000028672	Hmgcl	1211.80	1970.23	0.70	0.02714	Up
ENSMUSG00000020534	Shmt1	1010.82	1425.00	0.50	0.02719	Up
ENSMUSG00000028545	Bend5	502.78	801.83	0.67	0.02719	Up
ENSMUSG00000091952	Gm17709	133.44	301.88	1.18	0.0273	Up
ENSMUSG00000034613	Ppm1h	1928.62	2527.83	0.39	0.02764	Up
ENSMUSG00000002204	Napsa	19368.27	25527.28	0.40	0.02776	Up
ENSMUSG00000045636	Mtus1	1736.68	2481.97	0.52	0.02812	Up
ENSMUSG00000009145	Dqx1	185.47	260.12	0.49	0.0282	Up
ENSMUSG00000042428	Mgat3	1368.12	2345.47	0.78	0.0285	Up
ENSMUSG00000090165	Ugt1a10	123.10	239.04	0.96	0.02884	Up
ENSMUSG00000036534	Slc38a7	619.11	840.85	0.44	0.02918	Up
ENSMUSG00000021420	Fars2	745.84	1028.85	0.46	0.02927	Up
ENSMUSG00000027605	Acss2	2569.44	3866.19	0.59	0.02935	Up

ENSMUSG00000033735	Spr	2830.01	3896.03	0.46	0.02935	Up
ENSMUSG00000023707	Ogfod2	565.21	745.42	0.40	0.02941	Up
ENSMUSG00000090145	Ugt1a6b	18.90	58.90	1.64	0.02963	Up
ENSMUSG00000023031	Cela1	208.56	453.77	1.12	0.02964	Up
ENSMUSG00000021577	Sdha	8764.24	11974.94	0.45	0.02985	Up
ENSMUSG00000063428	Ddo	965.77	1284.05	0.41	0.02985	Up
ENSMUSG00000072949	Acot1	1525.50	2234.01	0.55	0.03014	Up
ENSMUSG00000027875	Hmgcs2	61.17	186.48	1.61	0.03039	Up
ENSMUSG00000036199	Ndufa13	2906.58	4156.02	0.52	0.03099	Up
ENSMUSG00000055435	Maf	2888.94	3857.77	0.42	0.03127	Up
ENSMUSG00000064254	Ethe1	1862.39	2709.41	0.54	0.03163	Up
ENSMUSG00000022571	Pycrl	887.40	1303.90	0.56	0.03174	Up
ENSMUSG00000018574	Acadvl	1826.74	2841.26	0.64	0.03175	Up
ENSMUSG00000054733	Msra	2858.92	4433.82	0.63	0.03175	Up
ENSMUSG00000022679	Mpv17l	9401.69	18484.50	0.98	0.03195	Up
ENSMUSG00000021982	Cdadc1	676.02	868.33	0.36	0.03221	Up
ENSMUSG00000021226	Acot2	570.81	807.18	0.50	0.03258	Up
ENSMUSG00000020329	Polrmt	895.16	1164.43	0.38	0.0327	Up
ENSMUSG00000028032	Papss1	2337.99	3558.72	0.61	0.0334	Up
ENSMUSG00000038195	Rilp	155.13	238.02	0.62	0.03372	Up
ENSMUSG00000015474	Ppt2	810.65	1349.66	0.74	0.03388	Up
ENSMUSG00000020621	Rdh14	478.05	639.18	0.42	0.03408	Up
ENSMUSG00000027893	Ahcyl1	4789.64	6000.30	0.33	0.03408	Up
ENSMUSG00000032080	Apoa4	47.24	113.01	1.26	0.03462	Up

ENSMUSG00000025197	Cyp2c44	47.54	99.91	1.07	0.03466	Up
ENSMUSG00000027367	Stard7	1791.93	2415.05	0.43	0.03471	Up
ENSMUSG00000025781	Atp5c1	5427.68	7199.80	0.41	0.03478	Up
ENSMUSG00000018509	Cenpv	264.21	387.98	0.55	0.03511	Up
ENSMUSG00000051671	Coa6	650.32	887.96	0.45	0.03512	Up
ENSMUSG00000031090	Nadsyn1	489.76	676.10	0.47	0.03526	Up
ENSMUSG00000021228	Acot3	50.56	118.99	1.23	0.03543	Up
ENSMUSG00000025393	Atp5b	41606.50	58752.81	0.50	0.03558	Up
ENSMUSG00000022426	Josd1	1454.32	1931.13	0.41	0.0364	Up
ENSMUSG00000021003	Galc	2045.58	3092.59	0.60	0.03655	Up
ENSMUSG00000035936	Aldh5a1	520.01	940.03	0.85	0.03665	Up
ENSMUSG00000038497	Tmco3	1781.70	2817.61	0.66	0.03809	Up
ENSMUSG00000029553	Tfec	602.68	883.09	0.55	0.0381	Up
ENSMUSG00000042082	Arsb	1274.64	2350.81	0.88	0.0381	Up
ENSMUSG00000003072	Atp5d	6179.10	8603.75	0.48	0.03842	Up
ENSMUSG00000001155	Ftcd	600.01	1337.85	1.16	0.03939	Up
ENSMUSG00000019429	Ffar3	4.27	19.68	2.20	0.0396	Up
ENSMUSG00000027359	Slc27a2	26942.28	43253.36	0.68	0.04002	Up
ENSMUSG00000024795	Kif20b	388.88	733.68	0.92	0.04024	Up
ENSMUSG00000073838	Tufm	2695.36	3590.46	0.41	0.04037	Up
ENSMUSG00000004748	Mtfp1	217.31	354.04	0.70	0.04037	Up
ENSMUSG00000033917	Gde1	1704.89	2285.39	0.42	0.04061	Up
ENSMUSG00000080950	Gm7278	82.22	208.44	1.34	0.04061	Up
ENSMUSG00000020777	Acox1	12268.02	21600.77	0.82	0.04079	Up

ENSMUSG00000015599	Ttbk1	101.09	226.85	1.17	0.04092	Up
ENSMUSG00000036932	Aifm1	2357.26	3125.94	0.41	0.04109	Up
ENSMUSG00000015970	Chdh	6756.65	8831.93	0.39	0.04138	Up
ENSMUSG00000025428	Atp5a1	26379.21	35147.65	0.41	0.04138	Up
ENSMUSG00000026526	Fh1	2600.58	3291.61	0.34	0.04138	Up
ENSMUSG00000032314	Etfa	3858.76	5618.50	0.54	0.04185	Up
ENSMUSG00000038679	Trps1	1838.30	2770.55	0.59	0.04186	Up
ENSMUSG00000078515	Ddi2	2933.06	3866.78	0.40	0.04237	Up
ENSMUSG00000015357	Clpx	2370.94	3619.83	0.61	0.04248	Up
ENSMUSG00000028834	Trim63	55.52	141.41	1.35	0.04282	Up
ENSMUSG00000054434	Tmem120b	146.67	224.69	0.62	0.04326	Up
ENSMUSG00000039519	Cyp7b1	1858.55	2945.42	0.66	0.04332	Up
ENSMUSG00000011831	Evi5	1897.26	2475.07	0.38	0.04361	Up
ENSMUSG00000055137	Sugct	1255.58	1874.06	0.58	0.04361	Up
ENSMUSG00000046687	Gm5424	15.04	41.88	1.48	0.04418	Up
ENSMUSG00000038236	Hoxa7	663.77	869.50	0.39	0.04431	Up
ENSMUSG00000031994	Adamts8	26.63	77.30	1.54	0.04547	Up
ENSMUSG00000029401	Rilpl2	537.89	756.56	0.49	0.04617	Up
ENSMUSG00000058927	Gm10053	30.21	60.23	1.00	0.04617	Up
ENSMUSG00000015806	Qdpr	2228.63	3679.11	0.72	0.04637	Up
ENSMUSG00000037263	1700055N04Rik	55.12	96.62	0.81	0.04687	Up
ENSMUSG00000002059	Rab34	1183.62	1565.86	0.40	0.04695	Up
ENSMUSG00000033624	Pdpr	857.32	1140.06	0.41	0.04793	Up
ENSMUSG00000032357	Tinag	1779.99	2428.05	0.45	0.04838	Up

ENSMUSG00000021792	Fam213a	3612.47	5966.42	0.72	0.0488	Up
ENSMUSG00000025347	Mettl7b	2433.70	4451.64	0.87	0.04919	Up
ENSMUSG00000057230	Aak1	508.66	656.84	0.37	0.04919	Up
ENSMUSG00000021900	Btd	1282.96	1684.54	0.39	0.04928	Up
ENSMUSG00000039633	Lonrf1	223.15	342.62	0.62	0.04928	Up
ENSMUSG00000047412	Zbtb44	1261.30	1694.03	0.43	0.04966	Up
ENSMUSG00000024997	Prdx3	1984.71	2668.02	0.43	0.0498	Up
ENSMUSG00000045594	Glb1	1721.10	3034.38	0.82	0.04992	Up
ENSMUSG00000001249	Hpn	7434.26	11643.64	0.65	0.04996	Up
ENSMUSG00000057614	Gnai1	2353.73	3356.60	0.51	0.04996	Up
ENSMUSG00000028270	Gbp2	26775.80	3073.27	-3.12	1.12E-14	Down
ENSMUSG00000040264	Gbp5	7326.03	1098.45	-2.74	1.12E-14	Down
ENSMUSG00000078922	Tgtp1	8540.94	1132.54	-2.91	6.97E-13	Down
ENSMUSG00000079343	C1s2	359.23	76.78	-2.23	7.38E-13	Down
ENSMUSG00000034438	Gbp8	1898.37	294.73	-2.69	9.73E-13	Down
ENSMUSG00000029298	Gbp9	2684.56	608.03	-2.14	2.85E-12	Down
ENSMUSG00000020826	Nos2	1268.59	138.16	-3.20	4.63E-12	Down
ENSMUSG00000025887	Casp12	994.73	358.22	-1.47	1.01E-11	Down
ENSMUSG00000028268	Gbp3	13173.91	3019.45	-2.13	3.76E-11	Down
ENSMUSG00000040253	Gbp7	9143.70	2021.15	-2.18	2.08E-10	Down
ENSMUSG00000034422	Parp14	8580.91	2398.35	-1.84	4.27E-10	Down
ENSMUSG00000078920	Ifi47	5367.83	683.39	-2.97	4.90E-10	Down
ENSMUSG00000037321	Tap1	6479.12	1371.59	-2.24	1.17E-09	Down
ENSMUSG00000002847	Pla1a	844.32	431.66	-0.97	1.48E-09	Down

ENSMUSG00000038301	Snx10	2421.02	690.22	-1.81	2.29E-09	Down
ENSMUSG00000018899	Irf1	7985.02	1812.45	-2.14	1.05E-08	Down
ENSMUSG00000027514	Zbp1	2227.60	301.05	-2.89	1.13E-08	Down
ENSMUSG00000002578	Ikzf4	225.82	97.76	-1.21	1.20E-07	Down
ENSMUSG00000041773	Enc1	1458.43	624.55	-1.22	1.50E-07	Down
ENSMUSG00000039899	Fgl2	2124.97	395.63	-2.43	1.69E-07	Down
ENSMUSG00000038521	C1s1	4663.61	1147.11	-2.02	3.35E-07	Down
ENSMUSG00000033538	Casp4	829.72	296.72	-1.48	3.51E-07	Down
ENSMUSG00000096727	Psmb9	4325.39	1008.32	-2.10	8.20E-07	Down
ENSMUSG00000027078	Ube2l6	2170.31	628.55	-1.79	2.75E-06	Down
ENSMUSG00000006782	Cnp	911.77	575.02	-0.67	2.94E-06	Down
ENSMUSG00000090958	Lrrc32	906.36	514.04	-0.82	3.01E-06	Down
ENSMUSG00000026536	Mnda	360.73	111.36	-1.70	3.06E-06	Down
ENSMUSG00000000957	Mmp14	2359.63	1241.29	-0.93	4.17E-06	Down
ENSMUSG00000032596	Uba7	1133.42	446.00	-1.35	4.17E-06	Down
ENSMUSG00000031897	Psmb10	4997.12	1721.05	-1.54	4.23E-06	Down
ENSMUSG00000024338	Psmb8	4521.36	1285.97	-1.81	6.85E-06	Down
ENSMUSG00000024349	Tmem173	1222.94	552.46	-1.15	1.33E-05	Down
ENSMUSG00000022575	Gsdmd	983.43	400.30	-1.30	1.69E-05	Down
ENSMUSG00000016283	H2-M2	495.02	137.11	-1.85	1.89E-05	Down
ENSMUSG00000055172	C1ra	3239.15	1145.22	-1.50	1.93E-05	Down
ENSMUSG00000002602	Axl	3221.24	1867.25	-0.79	2.10E-05	Down
ENSMUSG00000050370	Ch25h	251.48	73.80	-1.77	2.69E-05	Down
ENSMUSG00000085977	Gm5970	197.85	14.89	-3.73	3.12E-05	Down

ENSMUSG00000029798	Herc6	2526.48	728.39	-1.79	3.72E-05	Down
ENSMUSG00000058163	Gm5431	323.56	66.13	-2.29	3.85E-05	Down
ENSMUSG00000069892	9930111J21Rik2	605.44	237.32	-1.35	3.85E-05	Down
ENSMUSG00000038507	Parp12	2448.50	1015.34	-1.27	5.95E-05	Down
ENSMUSG00000060675	Pla2g16	2555.94	751.39	-1.77	6.12E-05	Down
ENSMUSG00000079363	Gbp4	9888.81	652.81	-3.92	6.12E-05	Down
ENSMUSG00000023903	Mmp25	121.17	23.03	-2.40	6.20E-05	Down
ENSMUSG00000024066	Xdh	2680.97	825.66	-1.70	9.25E-05	Down
ENSMUSG00000024014	Pim1	978.66	451.79	-1.12	0.0001	Down
ENSMUSG00000028789	Adc	451.36	274.79	-0.72	0.00014	Down
ENSMUSG00000019699	Akt3	951.25	452.47	-1.07	0.00015	Down
ENSMUSG00000035929	H2-Q4	4619.43	1961.31	-1.24	0.00017	Down
ENSMUSG00000073490	AI607873	701.25	294.30	-1.25	0.00019	Down
ENSMUSG00000027366	Sppl2a	5841.22	4036.09	-0.53	0.00019	Down
ENSMUSG00000025044	Msr1	499.16	172.33	-1.53	0.0002	Down
ENSMUSG00000018865	Sult4a1	610.41	313.32	-0.96	0.0002	Down
ENSMUSG00000024737	Slc15a3	641.59	266.14	-1.27	0.00021	Down
ENSMUSG00000000409	Lck	276.32	107.52	-1.36	0.00024	Down
ENSMUSG00000032294	Pkm	12304.87	8181.56	-0.59	0.00024	Down
ENSMUSG00000040296	Ddx58	2339.25	1417.84	-0.72	0.00026	Down
ENSMUSG00000063286	Gm8995	1047.16	317.95	-1.72	0.00027	Down
ENSMUSG00000046718	Bst2	6739.02	2535.98	-1.41	0.00032	Down
ENSMUSG00000025888	Casp1	253.24	79.10	-1.68	0.00032	Down
ENSMUSG00000024659	Anxa1	1181.18	845.32	-0.48	0.00034	Down

ENSMUSG00000025076	Casp7	1204.33	614.90	-0.97	0.00034	Down
ENSMUSG00000034205	Loxl2	1029.62	702.97	-0.55	0.00035	Down
ENSMUSG00000032661	Oas3	377.96	72.87	-2.37	0.00037	Down
ENSMUSG00000030560	Ctsc	5033.76	2284.64	-1.14	0.00038	Down
ENSMUSG00000027639	Samhd1	4241.46	1221.47	-1.80	0.00043	Down
ENSMUSG00000002458	Rgs19	530.16	355.23	-0.58	0.00043	Down
ENSMUSG00000012428	Steap4	464.58	199.51	-1.22	0.00047	Down
ENSMUSG00000075010	AW112010	1859.24	555.29	-1.74	0.00058	Down
ENSMUSG00000023132	Gzma	221.57	41.51	-2.42	0.00063	Down
ENSMUSG00000056144	Trim34a	141.97	65.09	-1.13	0.00063	Down
ENSMUSG00000032487	Ptgs2	113.14	33.16	-1.77	0.00065	Down
ENSMUSG00000038642	Ctss	5512.86	2340.55	-1.24	0.00065	Down
ENSMUSG00000053318	Slamf8	341.34	86.40	-1.98	0.00065	Down
ENSMUSG00000021094	Dhrs7	556.35	361.37	-0.62	0.00077	Down
ENSMUSG00000020407	Upp1	1018.56	300.28	-1.76	0.00081	Down
ENSMUSG00000078606	Gm4070	1181.16	329.13	-1.84	0.00081	Down
ENSMUSG00000032271	Nnmt	436.18	171.77	-1.34	0.00082	Down
ENSMUSG00000022221	Ripk3	267.44	148.97	-0.84	0.00089	Down
ENSMUSG00000052776	Oas1a	927.39	340.52	-1.45	0.0009	Down
ENSMUSG00000092517	Art2a-ps	118.33	35.78	-1.73	0.00118	Down
ENSMUSG00000053024	Cntn2	156.64	34.31	-2.19	0.00128	Down
ENSMUSG00000073409	H2-Q6	2692.64	1130.14	-1.25	0.00129	Down
ENSMUSG00000023043	Krt18	3166.52	2076.01	-0.61	0.00131	Down
ENSMUSG00000025355	Mmp19	107.82	50.00	-1.11	0.00154	Down

ENSMUSG00000038352	Arl5c	175.49	50.57	-1.79	0.0016	Down
ENSMUSG00000039236	Isg20	416.41	142.21	-1.55	0.0016	Down
ENSMUSG00000069874	Irgm2	9842.79	1613.58	-2.61	0.0016	Down
ENSMUSG00000021583	Erap1	3462.51	2007.25	-0.79	0.00168	Down
ENSMUSG00000015947	Fcgr1	601.53	258.31	-1.22	0.00182	Down
ENSMUSG00000098470	C1rb	122.99	38.90	-1.66	0.00191	Down
ENSMUSG00000057143	Trim12c	1557.42	554.18	-1.49	0.00224	Down
ENSMUSG00000022367	Has2	52.22	17.24	-1.60	0.00245	Down
ENSMUSG00000041323	Ak7	245.63	99.95	-1.30	0.0025	Down
ENSMUSG00000086513	9130208D14Rik	115.40	36.51	-1.66	0.00254	Down
ENSMUSG00000012519	Mlkl	639.62	238.17	-1.43	0.00256	Down
ENSMUSG00000031805	Jak3	1236.56	854.27	-0.53	0.00264	Down
ENSMUSG00000028843	Sh3bgrl3	1620.94	1034.58	-0.65	0.00288	Down
ENSMUSG00000000682	Cd52	1329.51	524.67	-1.34	0.00305	Down
ENSMUSG00000034118	Tpst1	585.84	249.95	-1.23	0.00306	Down
ENSMUSG00000078502	Gm13212	234.78	84.45	-1.48	0.00311	Down
ENSMUSG00000003283	Hck	621.34	244.09	-1.35	0.00356	Down
ENSMUSG00000017830	Dhx58	440.04	151.09	-1.54	0.00359	Down
ENSMUSG00000066861	Oas1g	282.73	110.31	-1.36	0.00363	Down
ENSMUSG00000082292	Gm12250	1466.89	129.57	-3.50	0.00379	Down
ENSMUSG00000035273	Hpse	288.52	92.00	-1.65	0.00379	Down
ENSMUSG00000027580	Helz2	2082.46	1141.38	-0.87	0.00391	Down
ENSMUSG00000051022	Hs3st1	177.44	92.62	-0.94	0.00397	Down
ENSMUSG00000073555	Gm4951	1074.73	115.53	-3.22	0.00435	Down

ENSMUSG00000024401	Tnf	199.32	69.76	-1.51	0.00482	Down
ENSMUSG00000024691	Fam111a	531.11	272.88	-0.96	0.00497	Down
ENSMUSG00000069793	Slfn9	392.00	192.14	-1.03	0.00499	Down
ENSMUSG00000035105	Egln3	598.51	374.55	-0.68	0.00507	Down
ENSMUSG00000063388	BC023105	933.28	99.78	-3.23	0.0052	Down
ENSMUSG00000054404	Slfn5	1154.27	572.58	-1.01	0.00526	Down
ENSMUSG00000022673	Mcm4	688.21	465.80	-0.56	0.00535	Down
ENSMUSG00000032038	St3gal4	496.10	345.93	-0.52	0.00566	Down
ENSMUSG00000024087	Cyp1b1	1822.29	1150.93	-0.66	0.00636	Down
ENSMUSG00000029605	Oas1b	183.68	82.48	-1.16	0.00737	Down
ENSMUSG00000026395	Ptprc	2145.18	986.01	-1.12	0.0075	Down
ENSMUSG00000058624	Gda	708.78	395.89	-0.84	0.00752	Down
ENSMUSG00000022378	Fam49b	1238.47	695.90	-0.83	0.00769	Down
ENSMUSG00000031722	Hp	302.69	119.03	-1.35	0.00784	Down
ENSMUSG00000069893	9930111J21Rik1	100.39	36.77	-1.45	0.00842	Down
ENSMUSG00000023961	Enpp4	1149.95	822.58	-0.48	0.00845	Down
ENSMUSG00000034573	Ptpn13	9085.48	5622.74	-0.69	0.00849	Down
ENSMUSG00000034266	Batf	155.21	71.33	-1.12	0.00913	Down
ENSMUSG00000063234	Gpr84	71.91	13.01	-2.47	0.0104	Down
ENSMUSG00000031659	Adcy7	650.70	373.92	-0.80	0.01049	Down
ENSMUSG00000026896	Ifih1	1235.73	581.23	-1.09	0.01059	Down
ENSMUSG00000031328	Flna	8909.97	6515.71	-0.45	0.01076	Down
ENSMUSG00000032089	Il10ra	718.25	349.24	-1.04	0.01141	Down
ENSMUSG00000029570	Lfng	603.15	357.85	-0.75	0.01147	Down

ENSMUSG00000033392	Clasp2	898.89	690.13	-0.38	0.01189	Down
ENSMUSG00000026042	Col5a2	1849.64	1059.46	-0.80	0.01215	Down
ENSMUSG00000028864	Hgf	156.31	94.48	-0.73	0.01258	Down
ENSMUSG00000039994	Timeless	316.48	218.29	-0.54	0.0132	Down
ENSMUSG00000049225	Pdp1	524.76	346.01	-0.60	0.01418	Down
ENSMUSG00000079445	B3gnt7	185.66	116.86	-0.67	0.01419	Down
ENSMUSG00000035547	Capn5	1014.17	714.09	-0.51	0.01451	Down
ENSMUSG00000029923	Rab19	176.66	85.31	-1.05	0.0147	Down
ENSMUSG00000032436	Cmtm7	343.20	188.04	-0.87	0.01499	Down
ENSMUSG00000022048	Dpysl2	525.75	346.52	-0.60	0.01512	Down
ENSMUSG00000028954	Nub1	1433.59	1024.14	-0.49	0.01531	Down
ENSMUSG00000035208	Slfn8	271.21	138.02	-0.97	0.01539	Down
ENSMUSG00000000486	01-9	179.68	97.39	-0.88	0.01563	Down
ENSMUSG00000018340	Anxa6	3451.51	2577.65	-0.42	0.01599	Down
ENSMUSG00000020357	Flt4	801.29	529.88	-0.60	0.01642	Down
ENSMUSG00000051236	Msrb3	487.95	342.12	-0.51	0.0165	Down
ENSMUSG00000029826	Zc3hav1	1937.87	1284.59	-0.59	0.0173	Down
ENSMUSG00000019139	Isyna1	2684.04	2141.19	-0.33	0.01773	Down
ENSMUSG00000052889	Prkcb	148.14	77.62	-0.93	0.01796	Down
ENSMUSG00000015143	Actn1	6485.14	4073.28	-0.67	0.018	Down
ENSMUSG00000024789	Jak2	2754.56	1847.59	-0.58	0.01828	Down
ENSMUSG00000051379	Flrt3	134.98	76.04	-0.83	0.01862	Down
ENSMUSG00000006360	Crip1	859.80	509.05	-0.76	0.01953	Down
ENSMUSG00000030137	Tuba8	83.23	34.11	-1.29	0.01953	Down

ENSMUSG00000039981	Zc3h12d	127.33	53.18	-1.26	0.01992	Down
ENSMUSG00000033565	Rbfox2	1528.95	1109.17	-0.46	0.02008	Down
ENSMUSG00000027009	Itga4	406.14	175.06	-1.21	0.02048	Down
ENSMUSG00000074622	Mafb	707.83	496.60	-0.51	0.02048	Down
ENSMUSG00000029084	Cd38	361.67	208.42	-0.80	0.02086	Down
ENSMUSG00000025001	Hells	322.56	210.68	-0.61	0.02093	Down
ENSMUSG00000000386	Mx1	266.46	113.95	-1.23	0.02121	Down
ENSMUSG00000031627	Irf2	1243.09	825.04	-0.59	0.02151	Down
ENSMUSG00000000127	Fer	356.65	243.70	-0.55	0.0216	Down
ENSMUSG00000026728	Vim	5863.78	3413.38	-0.78	0.02212	Down
ENSMUSG00000079057	Cyp4v3	694.58	459.53	-0.60	0.02212	Down
ENSMUSG00000029413	Naaa	1352.57	597.26	-1.18	0.02238	Down
ENSMUSG00000079442	St6galnac4	281.15	164.59	-0.77	0.02239	Down
ENSMUSG00000027293	Ehd4	2248.77	1745.20	-0.37	0.02272	Down
ENSMUSG00000041642	Kif21b	198.94	126.06	-0.66	0.02272	Down
ENSMUSG00000024910	Ctsw	128.61	63.88	-1.01	0.0234	Down
ENSMUSG00000039264	Gimap3	88.20	43.16	-1.03	0.02372	Down
ENSMUSG00000023927	Satb1	117.48	62.59	-0.91	0.02416	Down
ENSMUSG00000040711	Sh3pxd2b	589.29	378.85	-0.64	0.02535	Down
ENSMUSG00000026094	Stk17b	810.96	570.95	-0.51	0.02557	Down
ENSMUSG00000040809	Chil3	97.93	22.70	-2.11	0.0259	Down
ENSMUSG00000020092	Pald1	489.64	357.73	-0.45	0.02597	Down
ENSMUSG00000001444	Tbx21	56.48	17.91	-1.66	0.02647	Down
ENSMUSG00000024556	Me2	406.63	284.67	-0.51	0.02647	Down

ENSMUSG00000021262	Evl	540.35	365.58	-0.56	0.02764	Down
ENSMUSG00000049892	Rasd1	677.45	248.73	-1.45	0.02833	Down
ENSMUSG00000032093	Cd3e	67.97	31.54	-1.11	0.0284	Down
ENSMUSG00000060780	Lrrtm1	20.45	5.81	-1.81	0.02866	Down
ENSMUSG00000042842	Serpinb6b	2244.58	1455.88	-0.62	0.02878	Down
ENSMUSG00000065954	Tacc1	1922.53	1459.17	-0.40	0.02915	Down
ENSMUSG00000020143	Dock2	394.28	205.62	-0.94	0.02934	Down
ENSMUSG00000015653	Steap2	4347.98	3066.06	-0.50	0.02935	Down
ENSMUSG00000062488	I830012O16Rik	742.36	302.68	-1.29	0.02935	Down
ENSMUSG00000028364	Tnc	1257.55	639.68	-0.98	0.0297	Down
ENSMUSG00000031948	Kars	3226.42	2245.09	-0.52	0.02995	Down
ENSMUSG00000059248	09-9	2378.20	1817.00	-0.39	0.03095	Down
ENSMUSG00000033220	Rac2	889.83	488.30	-0.87	0.03174	Down
ENSMUSG00000032344	Mb21d1	106.18	58.42	-0.86	0.03183	Down
ENSMUSG00000052160	Pld4	1116.13	654.51	-0.77	0.03188	Down
ENSMUSG00000097804	Gm16685	112.48	53.66	-1.07	0.0327	Down
ENSMUSG00000006403	Adamts4	599.85	255.10	-1.23	0.03279	Down
ENSMUSG00000020134	Peli1	729.30	510.42	-0.51	0.03285	Down
ENSMUSG00000009585	Apobec3	485.76	304.05	-0.68	0.03292	Down
ENSMUSG00000055723	Rras2	2193.85	1640.30	-0.42	0.03344	Down
ENSMUSG00000001995	Sipa1l2	666.01	441.51	-0.59	0.03471	Down
ENSMUSG00000062296	Trank1	55.49	26.97	-1.04	0.03473	Down
ENSMUSG00000000693	Loxl3	288.31	188.23	-0.62	0.03478	Down
ENSMUSG00000091549	Gm6548	236.46	151.95	-0.64	0.03493	Down

ENSMUSG00000013584	Aldh1a2	1319.16	816.48	-0.69	0.03509	Down
ENSMUSG00000010529	Gm266	51.97	25.43	-1.03	0.03546	Down
ENSMUSG00000032091	Tmprss4	93.99	37.46	-1.33	0.03636	Down
ENSMUSG00000022952	Runx1	1265.03	715.72	-0.82	0.03654	Down
ENSMUSG00000020901	Pik3r5	290.70	165.90	-0.81	0.03707	Down
ENSMUSG00000027797	Dclk1	191.42	123.94	-0.63	0.03753	Down
ENSMUSG00000027540	Ptpn1	2677.99	1910.68	-0.49	0.03764	Down
ENSMUSG00000041135	Ripk2	504.01	326.13	-0.63	0.03952	Down
ENSMUSG00000003153	Slc2a3	77.42	39.21	-0.98	0.03971	Down
ENSMUSG00000004099	Dnmt1	1372.16	1092.98	-0.33	0.03973	Down
ENSMUSG00000056427	Slit3	376.20	233.59	-0.69	0.04002	Down
ENSMUSG00000015217	Hmgb3	2611.29	2009.89	-0.38	0.04028	Down
ENSMUSG00000038372	Gmds	889.34	627.85	-0.50	0.04028	Down
ENSMUSG00000075225	Ccdc162	93.98	53.27	-0.82	0.04092	Down
ENSMUSG00000024457	Trim26	1553.59	1163.84	-0.42	0.04138	Down
ENSMUSG00000058013	11-9	6373.16	4987.51	-0.35	0.04146	Down
ENSMUSG00000024054	Smchd1	1239.44	982.35	-0.34	0.04185	Down
ENSMUSG00000034463	Scara3	176.35	82.82	-1.09	0.04465	Down
ENSMUSG00000052949	Rnf157	121.09	68.67	-0.82	0.04467	Down
ENSMUSG00000054405	Dnajc8	1627.07	1342.01	-0.28	0.04469	Down
ENSMUSG00000026773	Pfkfb3	1067.97	586.38	-0.86	0.04569	Down
ENSMUSG00000057193	Slc44a2	1947.38	1457.55	-0.42	0.04689	Down
ENSMUSG00000024981	Acsl5	3363.28	2540.54	-0.40	0.04704	Down
ENSMUSG00000057335	Cep170	722.77	518.73	-0.48	0.04767	Down

ENSMUSG00000024300	Myo1f	636.97	304.86	-1.06	0.04913	Down
ENSMUSG00000039115	Itga9	943.40	665.63	-0.50	0.04937	Down
ENSMUSG00000031278	Acsl4	3467.69	2114.11	-0.71	0.04939	Down
ENSMUSG00000062421	Arf2	1024.78	830.14	-0.30	0.04942	Down
ENSMUSG00000070407	Hs3st3b1	188.13	108.21	-0.80	0.04966	Down

Supplementary Table 2. Significant differentially expression genes of metabolic process between pCAG-EGFP (control) and pCAG-APE1 injected kidneys after UUO

ID	Gene	EGFP_readcount	APE1_readcount	log2(fold change)	P value	Regulation
ENSMUSG00000048087	Gm4737	563.31	1734.26	1.62	1.22E-26	Up
ENSMUSG00000074639	BC089597	293.81	989.59	1.75	1.48E-09	Up
ENSMUSG00000078650	G6pc	543.06	2905.04	2.42	3.28E-09	Up
ENSMUSG00000040170	Fmo2	1626.91	3420.16	1.07	1.42E-08	Up
ENSMUSG00000010651	Acaa1b	821.47	3095.02	1.91	2.10E-08	Up
ENSMUSG00000020988	L2hgdh	1560.90	3078.10	0.98	2.36E-08	Up
ENSMUSG00000042102	Dmgdh	641.22	1433.22	1.16	5.08E-08	Up
ENSMUSG00000066097	Cyp2j11	897.69	2150.63	1.26	6.83E-08	Up
ENSMUSG00000010064	Slc38a3	661.18	1210.77	0.87	1.87E-07	Up
ENSMUSG00000040740	Slc25a34	298.64	646.45	1.11	1.87E-07	Up
ENSMUSG00000060961	Slc4a4	10983.10	17105.94	0.64	1.14E-06	Up
ENSMUSG00000037686	Aspg	526.14	875.70	0.73	1.14E-06	Up
ENSMUSG00000021416	Eci3	524.56	1232.06	1.23	1.39E-06	Up
ENSMUSG00000020051	Pah	2515.14	6869.66	1.45	2.08E-06	Up
ENSMUSG00000023044	Csad	2389.49	5670.93	1.25	2.75E-06	Up
ENSMUSG00000001467	Cyp51	2322.59	5128.56	1.14	4.77E-06	Up
ENSMUSG00000026348	Acmsd	483.48	1941.67	2.01	6.55E-06	Up
ENSMUSG00000030769	Slc5a11	425.01	992.34	1.22	7.42E-06	Up
ENSMUSG00000029772	Ahcyl2	3799.79	6617.62	0.80	1.24E-05	Up
ENSMUSG00000027070	Lrp2	15218.97	35951.69	1.24	1.80E-05	Up
ENSMUSG00000045316	Fahd1	1137.50	2095.48	0.88	2.96E-05	Up

ENSMUSG00000042251	Pm20d1	879.83	2537.17	1.53	3.21E-05	Up
ENSMUSG00000000673	Haao	730.58	2163.22	1.57	3.34E-05	Up
ENSMUSG00000028179	Cth	1499.77	2854.48	0.93	3.85E-05	Up
ENSMUSG00000061959	Ces1e	61.17	242.24	1.99	3.85E-05	Up
ENSMUSG00000022821	Hgd	1776.90	3791.33	1.09	4.71E-05	Up
ENSMUSG00000066072	Cyp4a10	718.32	2669.18	1.89	4.72E-05	Up
ENSMUSG00000025731	0610011F06Rik	1665.85	2841.50	0.77	5.46E-05	Up
ENSMUSG00000062410	Hsd3b3	236.10	557.28	1.24	6.00E-05	Up
ENSMUSG00000029695	Aass	2851.23	5885.26	1.05	6.20E-05	Up
ENSMUSG00000027187	Cat	5367.64	10842.39	1.01	7.12E-05	Up
ENSMUSG00000079261	Gm15217	382.11	804.66	1.07	7.68E-05	Up
ENSMUSG00000022708	Zbtb20	5233.04	7753.26	0.57	0.0000841	Up
ENSMUSG00000035878	Hykk	2153.48	5025.78	1.22	0.0000853	Up
ENSMUSG00000013611	Snx31	103.77	272.68	1.39	0.00011251	Up
ENSMUSG00000024781	Lipa	3209.62	5094.64	0.67	0.00011652	Up
ENSMUSG00000055373	Fut9	2945.78	6125.64	1.06	0.00014912	Up
ENSMUSG00000022235	Cmbl	639.13	1591.64	1.32	0.00014991	Up
ENSMUSG00000046598	Bdh1	1963.73	3991.21	1.02	0.00017367	Up
ENSMUSG00000027513	Pck1	4517.37	15664.93	1.79	0.00019114	Up
ENSMUSG00000024899	Papss2	1864.72	3403.00	0.87	0.00019323	Up
ENSMUSG00000042073	Abhd14b	955.38	1795.67	0.91	0.00020566	Up
ENSMUSG00000054619	Mettl7a1	3014.48	5945.04	0.98	0.00021477	Up
ENSMUSG00000040966	Slc22a2	2978.33	6107.90	1.04	0.00025932	Up
ENSMUSG00000029482	Aacs	1666.42	3072.08	0.88	0.00026884	Up

ENSMUSG00000028712	Cyp4a31	1481.13	2967.17	1.00	0.00029672	Up
ENSMUSG00000069805	Fbp1	4459.42	11235.58	1.33	0.00031181	Up
ENSMUSG00000058997	Vwa8	2657.09	4544.21	0.77	0.00032562	Up
ENSMUSG00000041052	Slc7a13	5014.82	15201.38	1.60	0.00033714	Up
ENSMUSG00000061578	Ksr2	198.64	470.68	1.24	0.00034478	Up
ENSMUSG00000038286	Bphl	1944.94	3714.24	0.93	0.00038278	Up
ENSMUSG00000080115	Mettl21b	56.74	124.46	1.13	0.00038278	Up
ENSMUSG00000003809	Gcdh	2349.78	4852.63	1.05	0.00042538	Up
ENSMUSG00000037440	Vnn1	654.08	1488.26	1.19	0.00044603	Up
ENSMUSG00000031445	Proz	40.60	136.50	1.75	0.0004974	Up
ENSMUSG00000040181	Fmo1	3193.36	5983.43	0.91	0.00050092	Up
ENSMUSG00000074254	Cyp2a4	2360.32	4804.48	1.03	0.00050277	Up
ENSMUSG00000026730	Pter	6305.31	9976.00	0.66	0.00051257	Up
ENSMUSG00000022546	Gpt	214.76	435.59	1.02	0.00053822	Up
ENSMUSG00000036395	Glb1l2	1694.72	2699.58	0.67	0.00053822	Up
ENSMUSG00000024713	Pcsk5	613.63	855.93	0.48	0.00054043	Up
ENSMUSG00000002769	Gnmt	114.10	277.79	1.28	0.00054282	Up
ENSMUSG00000038121	Fam210a	1662.81	2485.71	0.58	0.00054282	Up
ENSMUSG00000050097	Ces2b	83.78	270.29	1.69	0.00059388	Up
ENSMUSG00000020182	Ddc	422.50	826.35	0.97	0.00061579	Up
ENSMUSG00000001604	Tcea3	253.09	534.98	1.08	0.00063872	Up
ENSMUSG00000024131	Slc3a1	5282.51	8056.38	0.61	0.00065124	Up
ENSMUSG00000041426	Hibch	638.73	981.78	0.62	0.00066281	Up
ENSMUSG00000041757	Plekha6	2146.22	3048.70	0.51	0.00067926	Up

ENSMUSG00000060224	Pyroxd2	901.59	1561.33	0.79	0.00069812	Up
ENSMUSG00000023830	Igf2r	2662.31	4055.62	0.61	0.0007448	Up
ENSMUSG00000025728	Pigq	2529.95	3646.80	0.53	0.00074975	Up
ENSMUSG00000071669	Snx29	737.45	1178.44	0.68	0.00074975	Up
ENSMUSG00000028756	Pink1	3329.04	5251.32	0.66	0.00078393	Up
ENSMUSG00000022002	4930564B18Rik	9.49	39.53	2.06	0.00080716	Up
ENSMUSG00000035637	Grhpr	1841.53	3978.72	1.11	0.00081575	Up
ENSMUSG00000001755	Coasy	2505.36	4743.61	0.92	0.00088362	Up
ENSMUSG00000027610	Gss	3422.65	6133.76	0.84	0.00099074	Up
ENSMUSG00000027761	Aadac	757.94	1460.50	0.95	0.00099076	Up
ENSMUSG00000026200	Glb1l	1364.65	1973.14	0.53	0.0010452	Up
ENSMUSG00000021306	Gpr137b	1345.71	2609.30	0.96	0.0010551	Up
ENSMUSG00000032105	Pdzd3	976.02	2033.96	1.06	0.0010965	Up
ENSMUSG00000024978	Gpam	735.24	1515.33	1.04	0.0010982	Up
ENSMUSG00000024747	Aldh1a7	172.76	448.39	1.38	0.0011032	Up
ENSMUSG00000021079	Timm9	333.11	528.16	0.66	0.0011075	Up
ENSMUSG00000021846	Peli2	1065.20	1605.91	0.59	0.0011075	Up
ENSMUSG00000030972	Acsm5	532.07	1169.12	1.14	0.0011968	Up
ENSMUSG00000022742	Cpox	1837.99	3318.77	0.85	0.0012431	Up
ENSMUSG00000068086	Cyp2d9	717.61	1330.93	0.89	0.0012673	Up
ENSMUSG00000022797	Tfrc	1309.55	2164.09	0.72	0.0012779	Up
ENSMUSG00000029378	Areg	51.05	158.21	1.63	0.0013999	Up
ENSMUSG00000020186	Csrp2	1795.54	3054.73	0.77	0.0014014	Up
ENSMUSG00000039450	Dcxr	801.57	1686.98	1.07	0.0014169	Up

ENSMUSG00000029098	Acox3	1616.81	2772.56	0.78	0.0015206	Up
ENSMUSG00000075304	Sp5	122.54	247.61	1.01	0.0015876	Up
ENSMUSG00000015568	Lpl	3267.03	6418.91	0.97	0.0017087	Up
ENSMUSG00000025911	Adhfe1	660.16	1777.23	1.43	0.0017182	Up
ENSMUSG00000042797	Aqp11	123.69	306.74	1.31	0.0018325	Up
ENSMUSG00000042410	Agps	5282.40	9585.08	0.86	0.0019211	Up
ENSMUSG00000028518	Prkaa2	2086.92	3354.88	0.68	0.0019759	Up
ENSMUSG00000042371	Slc5a10	1568.02	2670.76	0.77	0.0021062	Up
ENSMUSG00000049152	Ugt3a2	7745.11	16647.14	1.10	0.0021464	Up
ENSMUSG00000027984	Hadh	4254.93	7334.95	0.79	0.0022437	Up
ENSMUSG00000030283	St8sia1	1565.00	2750.15	0.81	0.0022624	Up
ENSMUSG00000063730	Hsd3b2	1945.76	5020.18	1.37	0.0025249	Up
ENSMUSG00000030935	Acsm3	6417.75	14595.21	1.19	0.0025405	Up
ENSMUSG00000057228	Aadat	1434.73	3178.76	1.15	0.0025405	Up
ENSMUSG00000027227	Sord	5260.85	13447.71	1.35	0.0027493	Up
ENSMUSG00000016756	Cmah	688.04	1168.00	0.76	0.0027525	Up
ENSMUSG00000030747	Dgat2	1111.59	2338.12	1.07	0.0027759	Up
ENSMUSG00000028571	Cyp2j13	3719.90	7865.39	1.08	0.0028238	Up
ENSMUSG00000023019	Gpd1	1950.46	4503.32	1.21	0.0028719	Up
ENSMUSG00000024892	Pcx	3379.63	7472.55	1.14	0.0028719	Up
ENSMUSG00000031886	Ces2e	103.16	228.98	1.15	0.0028972	Up
ENSMUSG00000019989	Enpp3	1147.16	1801.11	0.65	0.0029113	Up
ENSMUSG00000035769	Xylb	3313.45	6406.80	0.95	0.0029436	Up
ENSMUSG00000033533	Acsm1	1009.93	2701.01	1.42	0.0029731	Up

ENSMUSG00000033965	Slc16a2	4642.07	6542.38	0.50	0.0031216	Up
ENSMUSG00000030945	Acsm2	30871.05	74598.18	1.27	0.003331	Up
ENSMUSG00000024437	Gm8615	156.70	288.41	0.88	0.0037279	Up
ENSMUSG00000025937	Lactb2	5702.42	12499.12	1.13	0.0038385	Up
ENSMUSG00000029802	Abcg2	4090.07	6408.96	0.65	0.0039666	Up
ENSMUSG00000038704	Aspdh	221.53	770.01	1.80	0.0039666	Up
ENSMUSG00000023262	Acy1	1979.75	3713.84	0.91	0.004083	Up
ENSMUSG00000020072	Pbld1	1030.15	2351.37	1.19	0.0043247	Up
ENSMUSG00000024900	Cpt1a	5017.74	7439.15	0.57	0.0044	Up
ENSMUSG00000026687	Aldh9a1	2931.43	5700.28	0.96	0.0044	Up
ENSMUSG00000086784	Isoc2a	855.56	1374.70	0.68	0.004431	Up
ENSMUSG00000022210	Dhrs4	1897.78	3991.70	1.07	0.0045459	Up
ENSMUSG00000025403	Shmt2	1676.77	3412.41	1.03	0.0045459	Up
ENSMUSG00000032081	Apoc3	105.83	287.26	1.44	0.0046333	Up
ENSMUSG00000075706	Gpx4	11153.01	15953.82	0.52	0.0047958	Up
ENSMUSG00000022751	Nit2	1086.15	1697.94	0.64	0.004933	Up
ENSMUSG00000027371	Fahd2a	493.42	896.16	0.86	0.004933	Up
ENSMUSG00000037762	Slc16a9	640.99	1335.36	1.06	0.0049545	Up
ENSMUSG00000022853	Ehhadh	5311.53	11921.88	1.17	0.0050225	Up
ENSMUSG00000060376	Bckdha	2748.01	4453.71	0.70	0.0050653	Up
ENSMUSG00000068587	Mgam	811.64	1321.64	0.70	0.0051925	Up
ENSMUSG00000027999	Pla2g12a	241.37	400.14	0.73	0.0052283	Up
ENSMUSG00000078695	Cisd3	520.97	856.31	0.72	0.0052582	Up
ENSMUSG00000003526	Prodh	1472.38	2456.30	0.74	0.0052633	Up

ENSMUSG00000017718	Afmid	97.44	216.84	1.15	0.0053352	Up
ENSMUSG00000090124	Ugt1a7c	254.69	473.64	0.90	0.0053549	Up
ENSMUSG00000062908	Acadm	9180.51	18162.82	0.98	0.0053853	Up
ENSMUSG00000030880	Polr3e	1090.75	1618.49	0.57	0.0056039	Up
ENSMUSG00000090150	Acad11	1011.81	1498.64	0.57	0.0056039	Up
ENSMUSG00000028307	Aldob	29575.02	53864.16	0.86	0.0056895	Up
ENSMUSG00000059406	Tmprss9	55.30	202.69	1.87	0.0057792	Up
ENSMUSG00000032047	Acat1	6646.06	12940.63	0.96	0.0057961	Up
ENSMUSG00000028603	Scp2	7605.59	12506.75	0.72	0.0059827	Up
ENSMUSG00000005547	Cyp2a5	235.69	776.03	1.72	0.0060723	Up
ENSMUSG00000025968	Ndufs1	2495.39	3587.65	0.52	0.0061177	Up
ENSMUSG00000055114	Anxa13	42.14	93.06	1.14	0.0065232	Up
ENSMUSG00000033863	Klf9	1881.09	3058.09	0.70	0.0065887	Up
ENSMUSG00000096917	2500002B13Rik	86.67	146.10	0.75	0.0071194	Up
ENSMUSG00000027809	Etfdh	2094.48	3012.69	0.52	0.0072609	Up
ENSMUSG00000002010	Idh3g	3170.76	4400.44	0.47	0.0072831	Up
ENSMUSG00000014361	Mertk	443.86	717.51	0.69	0.0073434	Up
ENSMUSG00000029162	Khk	4068.09	9150.84	1.17	0.007367	Up
ENSMUSG00000034371	Dak	4484.52	7479.86	0.74	0.007367	Up
ENSMUSG00000051065	Mb21d2	551.97	822.72	0.58	0.0074658	Up
ENSMUSG00000024921	Smarca2	1855.28	3602.52	0.96	0.0074798	Up
ENSMUSG00000021048	Mthfd1	1925.06	2850.89	0.57	0.00768	Up
ENSMUSG00000051510	Mafg	2217.90	2911.24	0.39	0.0082778	Up
ENSMUSG00000003949	Hlf	348.01	947.35	1.44	0.0084852	Up

ENSMUSG00000025453	Nnt	2591.73	3332.18	0.36	0.0085301	Up
ENSMUSG00000006378	Gcat	496.13	715.58	0.53	0.0086589	Up
ENSMUSG00000031969	Acad8	1030.65	1429.27	0.47	0.0088912	Up
ENSMUSG00000033488	BC026585	2798.30	4989.53	0.83	0.0089199	Up
ENSMUSG00000028405	Aco1	5640.33	7951.88	0.50	0.0089368	Up
ENSMUSG00000030800	Prss8	3676.36	6099.64	0.73	0.0092116	Up
ENSMUSG00000029556	Hnf1a	638.93	985.85	0.63	0.0092201	Up
ENSMUSG00000048578	Mlec	17381.96	26394.25	0.60	0.009551	Up
ENSMUSG00000058488	Kl	4236.74	7801.72	0.88	0.0095709	Up
ENSMUSG00000023068	Nus1	5686.40	9031.93	0.67	0.0097344	Up
ENSMUSG00000021263	Degs2	468.35	1288.57	1.46	0.0099395	Up
ENSMUSG00000021238	Aldh6a1	5785.77	10670.23	0.88	0.009966	Up
ENSMUSG00000015405	Ace2	2168.29	2998.89	0.47	0.010109	Up
ENSMUSG00000032418	Me1	6950.58	12139.00	0.80	0.010236	Up
ENSMUSG00000029735	Tpk1	1135.92	1925.21	0.76	0.010315	Up
ENSMUSG00000033453	Adamts15	266.10	535.14	1.01	0.010411	Up
ENSMUSG00000025903	Lypla1	3511.67	5780.32	0.72	0.010421	Up
ENSMUSG00000042647	Acad12	341.83	573.51	0.75	0.010478	Up
ENSMUSG00000000385	Tmprss2	2730.74	3595.64	0.40	0.010543	Up
ENSMUSG00000020098	Pcbd1	1599.67	2392.97	0.58	0.01076	Up
ENSMUSG00000019762	Iyd	415.59	745.05	0.84	0.01076	Up
ENSMUSG00000023921	Mut	2054.89	2920.16	0.51	0.0108	Up
ENSMUSG00000041650	Pcca	1644.35	2504.43	0.61	0.010881	Up
ENSMUSG00000026179	Pnkd	1568.61	2496.44	0.67	0.011115	Up

ENSMUSG00000020681	Ace	4729.51	8191.56	0.79	0.011215	Up
ENSMUSG00000044475	Ascc1	779.10	1268.51	0.70	0.011268	Up
ENSMUSG00000061906	Ugt2b38	4775.07	11172.86	1.23	0.01128	Up
ENSMUSG00000025486	Sirt3	557.06	1032.55	0.89	0.011292	Up
ENSMUSG00000017950	Hnf4a	10197.16	17481.97	0.78	0.011412	Up
ENSMUSG00000026958	Dpp7	2087.22	2890.83	0.47	0.011457	Up
ENSMUSG00000028973	Abcb8	1065.16	1515.44	0.51	0.011847	Up
ENSMUSG00000025209	Peo1	566.02	789.24	0.48	0.011872	Up
ENSMUSG00000072664	Ugt3a1	2545.47	5406.20	1.09	0.011903	Up
ENSMUSG00000025980	Hspd1	9413.53	14326.97	0.61	0.012255	Up
ENSMUSG00000006345	Ggt1	13741.98	27668.06	1.01	0.012322	Up
ENSMUSG00000032607	Amt	752.81	1093.39	0.54	0.012668	Up
ENSMUSG00000032540	Abhd5	668.37	904.71	0.44	0.013161	Up
ENSMUSG00000025545	Clybl	715.81	1199.15	0.74	0.013278	Up
ENSMUSG00000076441	Ass1	7858.12	14642.73	0.90	0.013387	Up
ENSMUSG00000024313	Mep1b	1108.66	2313.44	1.06	0.013525	Up
ENSMUSG00000029053	Prkcz	1289.98	1945.87	0.59	0.013722	Up
ENSMUSG00000055866	Per2	338.20	636.80	0.91	0.013767	Up
ENSMUSG00000053897	Slc39a8	938.61	1578.54	0.75	0.014063	Up
ENSMUSG00000030541	Idh2	4091.58	5510.32	0.43	0.014178	Up
ENSMUSG00000006313	Upk1a	90.17	218.22	1.28	0.014391	Up
ENSMUSG00000008226	Scrn3	558.87	896.58	0.68	0.01451	Up
ENSMUSG00000030630	Fah	3363.43	5563.68	0.73	0.014702	Up
ENSMUSG00000030522	Mtmr10	540.92	727.34	0.43	0.015309	Up

ENSMUSG00000026617	Bpnt1	2091.44	3499.09	0.74	0.015887	Up
ENSMUSG00000030102	Itpr1	5802.07	7595.91	0.39	0.015959	Up
ENSMUSG00000053644	Aldh7a1	3190.94	4994.45	0.65	0.016164	Up
ENSMUSG00000028088	Fmo5	633.72	1500.57	1.24	0.01683	Up
ENSMUSG00000024041	Cryaa	15.28	59.18	1.95	0.017298	Up
ENSMUSG00000027406	Idh3b	3610.63	4914.66	0.44	0.017298	Up
ENSMUSG00000004789	Dlst	5257.12	7734.95	0.56	0.017477	Up
ENSMUSG00000022445	Cyp2d26	75.64	165.54	1.13	0.01773	Up
ENSMUSG00000051169	Rpusd3	157.07	253.27	0.69	0.017733	Up
ENSMUSG00000015536	Mocs2	867.40	1381.71	0.67	0.017806	Up
ENSMUSG00000067916	Gm13139	1.52	36.48	4.59	0.017965	Up
ENSMUSG00000074064	Mlycd	821.03	1406.94	0.78	0.017965	Up
ENSMUSG00000093930	Hmgcs1	6247.96	9759.09	0.64	0.018176	Up
ENSMUSG00000024386	Proc	2153.82	4010.09	0.90	0.018604	Up
ENSMUSG00000020623	Map2k6	200.77	333.60	0.73	0.018622	Up
ENSMUSG00000041168	Lonp1	3362.54	4576.20	0.44	0.019002	Up
ENSMUSG00000016194	Hsd11b1	2840.86	5456.41	0.94	0.019516	Up
ENSMUSG00000029311	Hsd17b11	3248.70	6648.62	1.03	0.019526	Up
ENSMUSG00000037542	Aldh8a1	2740.57	5251.15	0.94	0.019594	Up
ENSMUSG00000003546	Klc4	1941.05	2857.75	0.56	0.019681	Up
ENSMUSG00000043681	Fam25c	33.32	82.72	1.31	0.019701	Up
ENSMUSG00000061838	Suclg2	4849.28	6713.32	0.47	0.020067	Up
ENSMUSG00000022186	Oxct1	10738.21	15963.74	0.57	0.020166	Up
ENSMUSG00000084854	Gm12678	109.20	301.31	1.46	0.020479	Up

ENSMUSG00000025059	Gyk	5900.44	10765.90	0.87	0.020986	Up
ENSMUSG00000090231	Cfb	288.72	457.96	0.67	0.021287	Up
ENSMUSG00000020774	Aspa	437.12	1035.25	1.24	0.021498	Up
ENSMUSG00000029455	Aldh2	9177.69	13133.74	0.52	0.021595	Up
ENSMUSG00000027332	Ivd	3460.35	5537.55	0.68	0.021695	Up
ENSMUSG00000084350	Znf41-ps	0.00	30.19	#DIV/0!	0.021769	Up
ENSMUSG00000052459	Atp6v1a	8181.27	11995.49	0.55	0.021903	Up
ENSMUSG00000052520	Cyp2j5	17214.83	25630.21	0.57	0.022112	Up
ENSMUSG00000023092	Fhl1	2345.52	4157.07	0.83	0.022138	Up
ENSMUSG00000041372	B4galnt3	616.77	902.31	0.55	0.022287	Up
ENSMUSG00000032098	Treh	1495.27	2988.46	1.00	0.02249	Up
ENSMUSG00000046814	Gchfr	141.69	294.82	1.06	0.022549	Up
ENSMUSG00000050645	Defb19	5.82	21.46	1.88	0.022712	Up
ENSMUSG00000010048	Ifrd2	1177.29	1969.06	0.74	0.022804	Up
ENSMUSG00000021322	Aoah	655.27	1148.91	0.81	0.023166	Up
ENSMUSG00000020664	Dld	4140.68	5492.60	0.41	0.02372	Up
ENSMUSG00000040658	Dnph1	276.11	550.42	1.00	0.023988	Up
ENSMUSG00000060227	Casc4	907.60	1576.01	0.80	0.024015	Up
ENSMUSG00000090817	Gm4450	1401.79	2430.78	0.79	0.024041	Up
ENSMUSG00000033610	Pank1	3457.62	6713.72	0.96	0.024161	Up
ENSMUSG00000021947	Cryl1	1608.45	3015.96	0.91	0.025042	Up
ENSMUSG00000020321	Mdh1	10424.82	15034.10	0.53	0.025209	Up
ENSMUSG00000056204	Pgpep1	2946.93	4003.12	0.44	0.02523	Up
ENSMUSG00000019767	Ccdc170	6.67	30.57	2.20	0.026144	Up

ENSMUSG00000025465	Echs1	2541.71	3633.81	0.52	0.026144	Up
ENSMUSG00000006575	Rundc3a	543.26	722.50	0.41	0.02647	Up
ENSMUSG00000021707	Dhfr	601.43	954.29	0.67	0.026673	Up
ENSMUSG00000048997	Atxn7l2	255.36	394.46	0.63	0.026673	Up
ENSMUSG00000086010	Gm15318	17.45	66.21	1.92	0.026744	Up
ENSMUSG00000031844	Hsd17b2	363.77	583.88	0.68	0.027107	Up
ENSMUSG00000014905	Dnajb9	1418.90	1807.31	0.35	0.027143	Up
ENSMUSG00000020534	Shmt1	1010.82	1425.00	0.50	0.027185	Up
ENSMUSG00000028545	Bend5	502.78	801.83	0.67	0.027185	Up
ENSMUSG00000039704	Lmbrd2	763.82	1094.22	0.52	0.027343	Up
ENSMUSG00000002204	Napsa	19368.27	25527.28	0.40	0.027764	Up
ENSMUSG00000045636	Mtus1	1736.68	2481.97	0.52	0.02812	Up
ENSMUSG00000042428	Mgat3	1368.12	2345.47	0.78	0.028499	Up
ENSMUSG00000090165	Ugt1a10	123.10	239.04	0.96	0.028842	Up
ENSMUSG00000036534	Slc38a7	619.11	840.85	0.44	0.029178	Up
ENSMUSG00000021420	Fars2	745.84	1028.85	0.46	0.029266	Up
ENSMUSG00000021779	Thrb	554.70	740.99	0.42	0.029294	Up
ENSMUSG00000027605	Acss2	2569.44	3866.19	0.59	0.029354	Up
ENSMUSG00000033735	Spr	2830.01	3896.03	0.46	0.029354	Up
ENSMUSG00000023707	Ogfod2	565.21	745.42	0.40	0.029407	Up
ENSMUSG00000090145	Ugt1a6b	18.90	58.90	1.64	0.029634	Up
ENSMUSG00000023031	Cela1	208.56	453.77	1.12	0.029642	Up
ENSMUSG00000021577	Sdha	8764.24	11974.94	0.45	0.029849	Up
ENSMUSG00000063428	Ddo	965.77	1284.05	0.41	0.029849	Up

ENSMUSG00000072949	Acot1	1525.50	2234.01	0.55	0.030143	Up
ENSMUSG00000025825	Iscu	2360.31	3051.89	0.37	0.030143	Up
ENSMUSG00000027875	Hmgcs2	61.17	186.48	1.61	0.030387	Up
ENSMUSG00000060981	Hist1h4h	154.05	241.09	0.65	0.030459	Up
ENSMUSG00000022477	Aco2	10744.29	17042.39	0.67	0.030648	Up
ENSMUSG00000036199	Ndufa13	2906.58	4156.02	0.52	0.030987	Up
ENSMUSG00000055435	Maf	2888.94	3857.77	0.42	0.031268	Up
ENSMUSG00000022571	Pycrl	887.40	1303.90	0.56	0.031741	Up
ENSMUSG00000018574	Acadvl	1826.74	2841.26	0.64	0.031745	Up
ENSMUSG00000054733	Msra	2858.92	4433.82	0.63	0.031745	Up
ENSMUSG00000022389	Tef	1377.71	2617.97	0.93	0.031826	Up
ENSMUSG00000022679	Mpv17l	9401.69	18484.50	0.98	0.03195	Up
ENSMUSG00000021226	Acot2	570.81	807.18	0.50	0.032579	Up
ENSMUSG00000020329	Polrmt	895.16	1164.43	0.38	0.032701	Up
ENSMUSG00000037071	Scd1	3144.94	4665.58	0.57	0.032704	Up
ENSMUSG00000025495	Ptdss2	1652.36	2045.78	0.31	0.032923	Up
ENSMUSG00000047963	Stbd1	635.62	891.99	0.49	0.033284	Up
ENSMUSG00000028032	Papss1	2337.99	3558.72	0.61	0.033396	Up
ENSMUSG00000015474	Ppt2	810.65	1349.66	0.74	0.033884	Up
ENSMUSG00000020621	Rdh14	478.05	639.18	0.42	0.034084	Up
ENSMUSG00000027893	Ahcyl1	4789.64	6000.30	0.33	0.034084	Up
ENSMUSG00000049971	Glt1d1	123.10	212.46	0.79	0.034508	Up
ENSMUSG00000070661	Rnf186	184.23	294.15	0.68	0.034551	Up
ENSMUSG00000032080	Apoa4	47.24	113.01	1.26	0.034622	Up

ENSMUSG00000025197	Cyp2c44	47.54	99.91	1.07	0.034663	Up
ENSMUSG00000027367	Stard7	1791.93	2415.05	0.43	0.034708	Up
ENSMUSG00000025781	Atp5c1	5427.68	7199.80	0.41	0.034783	Up
ENSMUSG00000018509	Cenpv	264.21	387.98	0.55	0.035106	Up
ENSMUSG00000031090	Nadsyn1	489.76	676.10	0.47	0.035263	Up
ENSMUSG00000021228	Acot3	50.56	118.99	1.23	0.035434	Up
ENSMUSG00000041653	Pnpla3	19.89	49.45	1.31	0.035948	Up
ENSMUSG00000022426	Josd1	1454.32	1931.13	0.41	0.036398	Up
ENSMUSG00000021003	Galc	2045.58	3092.59	0.60	0.036547	Up
ENSMUSG00000035936	Aldh5a1	520.01	940.03	0.85	0.036647	Up
ENSMUSG00000029553	Tfec	602.68	883.09	0.55	0.038095	Up
ENSMUSG00000042082	Arsb	1274.64	2350.81	0.88	0.038095	Up
ENSMUSG00000053040	Aph1c	631.66	954.27	0.60	0.038272	Up
ENSMUSG00000003072	Atp5d	6179.10	8603.75	0.48	0.038421	Up
ENSMUSG00000001155	Ftcd	600.01	1337.85	1.16	0.03939	Up
ENSMUSG00000019429	Ffar3	4.27	19.68	2.20	0.039603	Up
ENSMUSG00000027359	Slc27a2	26942.28	43253.36	0.68	0.040023	Up
ENSMUSG00000004748	Mtfp1	217.31	354.04	0.70	0.040372	Up
ENSMUSG00000033917	Gde1	1704.89	2285.39	0.42	0.040611	Up
ENSMUSG00000020777	Acox1	12268.02	21600.77	0.82	0.040786	Up
ENSMUSG00000015599	Ttbk1	101.09	226.85	1.17	0.040924	Up
ENSMUSG00000025812	Pard3	1405.28	1784.18	0.34	0.041061	Up
ENSMUSG00000036932	Aifm1	2357.26	3125.94	0.41	0.041091	Up
ENSMUSG00000015970	Chdh	6756.65	8831.93	0.39	0.041378	Up

ENSMUSG00000026526	Fh1	2600.58	3291.61	0.34	0.041378	Up
ENSMUSG00000032314	Etfa	3858.76	5618.50	0.54	0.041847	Up
ENSMUSG00000038679	Trps1	1838.30	2770.55	0.59	0.041857	Up
ENSMUSG00000078515	Ddi2	2933.06	3866.78	0.40	0.042366	Up
ENSMUSG00000015357	Clpx	2370.94	3619.83	0.61	0.04248	Up
ENSMUSG00000028834	Trim63	55.52	141.41	1.35	0.042821	Up
ENSMUSG00000071547	Nt5dc2	459.52	843.73	0.88	0.042821	Up
ENSMUSG00000020889	Nr1d1	168.89	417.64	1.31	0.043171	Up
ENSMUSG00000054434	Tmem120b	146.67	224.69	0.62	0.043262	Up
ENSMUSG00000039519	Cyp7b1	1858.55	2945.42	0.66	0.043322	Up
ENSMUSG00000011831	Evi5	1897.26	2475.07	0.38	0.043608	Up
ENSMUSG00000055137	Sugct	1255.58	1874.06	0.58	0.043608	Up
ENSMUSG00000046687	Gm5424	15.04	41.88	1.48	0.044183	Up
ENSMUSG00000038236	Hoxa7	663.77	869.50	0.39	0.044313	Up
ENSMUSG00000031994	Adamts8	26.63	77.30	1.54	0.045468	Up
ENSMUSG00000029401	Rilpl2	537.89	756.56	0.49	0.046166	Up
ENSMUSG00000015806	Qdpr	2228.63	3679.11	0.72	0.04637	Up
ENSMUSG00000022295	Atp6v1c1	2534.51	3248.52	0.36	0.046867	Up
ENSMUSG00000037263	1700055N04Rik	55.12	96.62	0.81	0.046867	Up
ENSMUSG00000047284	Neurl4	2058.68	2976.67	0.53	0.046867	Up
ENSMUSG00000033624	Pdpr	857.32	1140.06	0.41	0.047929	Up
ENSMUSG00000032652	Crebl2	718.29	957.68	0.41	0.048314	Up
ENSMUSG00000032357	Tinag	1779.99	2428.05	0.45	0.048384	Up
ENSMUSG00000021792	Fam213a	3612.47	5966.42	0.72	0.048801	Up

ENSMUSG00000025347	Mettl7b	2433.70	4451.64	0.87	0.049189	Up
ENSMUSG00000057230	Aak1	508.66	656.84	0.37	0.049189	Up
ENSMUSG00000039633	Lonrf1	223.15	342.62	0.62	0.049284	Up
ENSMUSG00000021900	Btd	1282.96	1684.54	0.39	0.049284	Up
ENSMUSG00000033726	Emx1	396.07	622.58	0.65	0.049367	Up
ENSMUSG00000047412	Zbtb44	1261.30	1694.03	0.43	0.049655	Up
ENSMUSG00000024997	Prdx3	1984.71	2668.02	0.43	0.049798	Up
ENSMUSG00000045594	Glb1	1721.10	3034.38	0.82	0.049919	Up
ENSMUSG00000059534	Uqcr10	3187.78	4531.91	0.51	0.049919	Up
ENSMUSG00000001249	Hpn	7434.26	11643.64	0.65	0.049957	Up
ENSMUSG00000028270	Gbp2	26775.80	3073.27	-3.12	1.12E-14	Down
ENSMUSG00000040264	Gbp5	7326.03	1098.45	-2.74	1.12E-14	Down
ENSMUSG00000078922	Tgtp1	8540.94	1132.54	-2.91	6.97E-13	Down
ENSMUSG00000079343	C1s2	359.23	76.78	-2.23	7.38E-13	Down
ENSMUSG00000034438	Gbp8	1898.37	294.73	-2.69	9.73E-13	Down
ENSMUSG00000020826	Nos2	1268.59	138.16	-3.20	4.63E-12	Down
ENSMUSG00000025887	Casp12	994.73	358.22	-1.47	1.01E-11	Down
ENSMUSG00000040253	Gbp7	9143.70	2021.15	-2.18	2.08E-10	Down
ENSMUSG00000034422	Parp14	8580.91	2398.35	-1.84	4.27E-10	Down
ENSMUSG00000078920	Ifi47	5367.83	683.39	-2.97	4.90E-10	Down
ENSMUSG00000037321	Tap1	6479.12	1371.59	-2.24	1.17E-09	Down
ENSMUSG00000027514	Zbp1	2227.60	301.05	-2.89	1.13E-08	Down
ENSMUSG00000041773	Enc1	1458.43	624.55	-1.22	1.50E-07	Down
ENSMUSG00000039899	Fgl2	2124.97	395.63	-2.43	1.69E-07	Down

ENSMUSG00000026980	Ly75	348.02	88.66	-1.97	1.95E-07	Down
ENSMUSG00000038521	C1s1	4663.61	1147.11	-2.02	3.35E-07	Down
ENSMUSG00000033538	Casp4	829.72	296.72	-1.48	3.51E-07	Down
ENSMUSG00000096727	Psmb9	4325.39	1008.32	-2.10	8.20E-07	Down
ENSMUSG00000073489	Ifi204	1359.20	342.13	-1.99	1.16E-06	Down
ENSMUSG00000030187	Klra2	269.77	51.69	-2.38	2.17E-06	Down
ENSMUSG00000027078	Ube2l6	2170.31	628.55	-1.79	2.75E-06	Down
ENSMUSG00000006782	Cnp	911.77	575.02	-0.67	2.94E-06	Down
ENSMUSG00000090958	Lrrc32	906.36	514.04	-0.82	3.01E-06	Down
ENSMUSG00000026536	Mnda	360.73	111.36	-1.70	3.06E-06	Down
ENSMUSG00000041515	Irf8	3962.63	1144.54	-1.79	3.77E-06	Down
ENSMUSG00000000957	Mmp14	2359.63	1241.29	-0.93	4.17E-06	Down
ENSMUSG00000032596	Uba7	1133.42	446.00	-1.35	4.17E-06	Down
ENSMUSG00000031897	Psmb10	4997.12	1721.05	-1.54	4.23E-06	Down
ENSMUSG00000022504	Ciita	3575.81	1013.47	-1.82	5.70E-06	Down
ENSMUSG00000024338	Psmb8	4521.36	1285.97	-1.81	6.85E-06	Down
ENSMUSG00000028459	Cd72	309.23	157.21	-0.98	1.09E-05	Down
ENSMUSG00000026452	Syt2	109.23	37.44	-1.54	1.13E-05	Down
ENSMUSG00000024349	Tmem173	1222.94	552.46	-1.15	1.33E-05	Down
ENSMUSG00000022575	Gsdmd	983.43	400.30	-1.30	1.69E-05	Down
ENSMUSG00000016283	H2-M2	495.02	137.11	-1.85	1.89E-05	Down
ENSMUSG00000055172	C1ra	3239.15	1145.22	-1.50	1.93E-05	Down
ENSMUSG00000002602	Axl	3221.24	1867.25	-0.79	2.10E-05	Down
ENSMUSG00000091649	Phf11b	450.88	123.54	-1.87	2.34E-05	Down

ENSMUSG00000050370	Ch25h	251.48	73.80	-1.77	2.69E-05	Down
ENSMUSG00000040033	Stat2	2407.02	911.18	-1.40	3.10E-05	Down
ENSMUSG00000085977	Gm5970	197.85	14.89	-3.73	3.12E-05	Down
ENSMUSG00000050014	Apol10b	193.59	20.68	-3.23	3.29E-05	Down
ENSMUSG00000058163	Gm5431	323.56	66.13	-2.29	3.85E-05	Down
ENSMUSG00000069892	9930111J21Rik2	605.44	237.32	-1.35	3.85E-05	Down
ENSMUSG00000023903	Mmp25	121.17	23.03	-2.40	6.20E-05	Down
ENSMUSG00000024066	Xdh	2680.97	825.66	-1.70	9.25E-05	Down
ENSMUSG00000024014	Pim1	978.66	451.79	-1.12	0.00010346	Down
ENSMUSG00000019699	Akt3	951.25	452.47	-1.07	0.00014739	Down
ENSMUSG00000035929	H2-Q4	4619.43	1961.31	-1.24	0.0001688	Down
ENSMUSG00000073490	AI607873	701.25	294.30	-1.25	0.00019114	Down
ENSMUSG00000021614	Vcan	550.41	209.40	-1.39	0.0001971	Down
ENSMUSG00000025044	Msr1	499.16	172.33	-1.53	0.00019882	Down
ENSMUSG00000042712	Wbp5	2831.30	2010.52	-0.49	0.00021248	Down
ENSMUSG00000030966	Trim21	590.74	234.33	-1.33	0.00022352	Down
ENSMUSG00000000409	Lck	276.32	107.52	-1.36	0.00023775	Down
ENSMUSG00000032294	Pkm	12304.87	8181.56	-0.59	0.00024119	Down
ENSMUSG00000046718	Bst2	6739.02	2535.98	-1.41	0.00032111	Down
ENSMUSG00000025888	Casp1	253.24	79.10	-1.68	0.00032174	Down
ENSMUSG00000024659	Anxa1	1181.18	845.32	-0.48	0.00033714	Down
ENSMUSG00000025076	Casp7	1204.33	614.90	-0.97	0.00033714	Down
ENSMUSG00000034205	Loxl2	1029.62	702.97	-0.55	0.00034939	Down
ENSMUSG00000032661	Oas3	377.96	72.87	-2.37	0.00037481	Down

ENSMUSG00000030560	Ctsc	5033.76	2284.64	-1.14	0.00038278	Down
ENSMUSG00000027639	Samhd1	4241.46	1221.47	-1.80	0.0004291	Down
ENSMUSG00000002458	Rgs19	530.16	355.23	-0.58	0.00043357	Down
ENSMUSG00000031596	Slc7a2	217.38	101.26	-1.10	0.00043822	Down
ENSMUSG00000012428	Steap4	464.58	199.51	-1.22	0.0004706	Down
ENSMUSG00000075010	AW112010	1859.24	555.29	-1.74	0.00058237	Down
ENSMUSG00000049103	Ccr2	2600.54	1097.18	-1.25	0.00059344	Down
ENSMUSG00000023132	Gzma	221.57	41.51	-2.42	0.00063092	Down
ENSMUSG00000056144	Trim34a	141.97	65.09	-1.13	0.00063092	Down
ENSMUSG00000032487	Ptgs2	113.14	33.16	-1.77	0.00065059	Down
ENSMUSG00000038642	Ctss	5512.86	2340.55	-1.24	0.00065059	Down
ENSMUSG00000053318	Slamf8	341.34	86.40	-1.98	0.00065115	Down
ENSMUSG00000028037	Ifi44	768.32	319.14	-1.27	0.00074975	Down
ENSMUSG00000021094	Dhrs7	556.35	361.37	-0.62	0.00077457	Down
ENSMUSG00000020407	Upp1	1018.56	300.28	-1.76	0.00080798	Down
ENSMUSG00000022221	Ripk3	267.44	148.97	-0.84	0.00089228	Down
ENSMUSG00000030786	Itgam	679.17	372.62	-0.87	0.0010264	Down
ENSMUSG00000006930	Hap1	458.82	241.66	-0.92	0.0010808	Down
ENSMUSG00000092517	Art2a-ps	118.33	35.78	-1.73	0.0011787	Down
ENSMUSG00000053024	Cntn2	156.64	34.31	-2.19	0.0012779	Down
ENSMUSG00000073409	H2-Q6	2692.64	1130.14	-1.25	0.0012945	Down
ENSMUSG00000023043	Krt18	3166.52	2076.01	-0.61	0.0013123	Down
ENSMUSG00000019907	Ppp1r12a	1755.12	1163.39	-0.59	0.0014752	Down
ENSMUSG00000025355	Mmp19	107.82	50.00	-1.11	0.0015418	Down

ENSMUSG00000038352	Arl5c	175.49	50.57	-1.79	0.0015997	Down
ENSMUSG00000039236	Isg20	416.41	142.21	-1.55	0.0015997	Down
ENSMUSG00000021583	Erap1	3462.51	2007.25	-0.79	0.0016807	Down
ENSMUSG00000015947	Fcgr1	601.53	258.31	-1.22	0.0018195	Down
ENSMUSG00000098470	C1rb	122.99	38.90	-1.66	0.0019102	Down
ENSMUSG00000034595	Ppp1r18	1013.95	669.49	-0.60	0.002007	Down
ENSMUSG00000051413	Plagl2	558.20	355.22	-0.65	0.0020354	Down
ENSMUSG00000022440	C1qtnf6	627.05	396.06	-0.66	0.0021689	Down
ENSMUSG00000057143	Trim12c	1557.42	554.18	-1.49	0.0022406	Down
ENSMUSG00000041323	Ak7	245.63	99.95	-1.30	0.0025028	Down
ENSMUSG00000012519	Mlkl	639.62	238.17	-1.43	0.0025576	Down
ENSMUSG00000031805	Jak3	1236.56	854.27	-0.53	0.0026443	Down
ENSMUSG00000000682	Cd52	1329.51	524.67	-1.34	0.0030533	Down
ENSMUSG00000028071	Sh2d2a	76.83	21.98	-1.81	0.0030621	Down
ENSMUSG00000071714	Csf2rb2	291.02	118.60	-1.29	0.0030621	Down
ENSMUSG00000078502	Gm13212	234.78	84.45	-1.48	0.0031064	Down
ENSMUSG00000027460	Angpt4	89.47	26.95	-1.73	0.003331	Down
ENSMUSG00000021958	Pinx1	391.36	251.04	-0.64	0.0033723	Down
ENSMUSG00000037820	Tgm2	14084.13	8676.62	-0.70	0.0033723	Down
ENSMUSG00000028466	Creb3	2128.49	1436.68	-0.57	0.0033859	Down
ENSMUSG00000003283	Hck	621.34	244.09	-1.35	0.0035631	Down
ENSMUSG00000082292	Gm12250	1466.89	129.57	-3.50	0.0037903	Down
ENSMUSG00000073555	Gm4951	1074.73	115.53	-3.22	0.0043494	Down
ENSMUSG00000040747	Cd53	831.44	439.11	-0.92	0.0044542	Down

ENSMUSG00000023034	Nr4a1	233.39	127.78	-0.87	0.0047054	Down
ENSMUSG00000024401	Tnf	199.32	69.76	-1.51	0.0048196	Down
ENSMUSG00000024691	Fam111a	531.11	272.88	-0.96	0.0049702	Down
ENSMUSG00000026630	Batf3	138.29	55.48	-1.32	0.0050253	Down
ENSMUSG00000035105	Egln3	598.51	374.55	-0.68	0.005072	Down
ENSMUSG00000063388	BC023105	933.28	99.78	-3.23	0.0052024	Down
ENSMUSG00000022673	Mcm4	688.21	465.80	-0.56	0.0053534	Down
ENSMUSG00000032038	St3gal4	496.10	345.93	-0.52	0.0056641	Down
ENSMUSG00000024087	Cyp1b1	1822.29	1150.93	-0.66	0.0063571	Down
ENSMUSG00000093661	Eif4e3	996.26	502.23	-0.99	0.0068286	Down
ENSMUSG00000026395	Ptprc	2145.18	986.01	-1.12	0.0075049	Down
ENSMUSG00000031722	Hp	302.69	119.03	-1.35	0.0078376	Down
ENSMUSG00000069893	9930111J21Rik1	100.39	36.77	-1.45	0.008419	Down
ENSMUSG00000023961	Enpp4	1149.95	822.58	-0.48	0.008451	Down
ENSMUSG00000034573	Ptpn13	9085.48	5622.74	-0.69	0.0084878	Down
ENSMUSG00000031103	Elf4	558.08	360.34	-0.63	0.008838	Down
ENSMUSG00000034266	Batf	155.21	71.33	-1.12	0.0091303	Down
ENSMUSG00000099241	Gm18852	36.44	5.90	-2.63	0.0091735	Down
ENSMUSG00000090125	Pou3f1	38.44	8.77	-2.13	0.010315	Down
ENSMUSG00000063234	Gpr84	71.91	13.01	-2.47	0.010403	Down
ENSMUSG00000031659	Adcy7	650.70	373.92	-0.80	0.010487	Down
ENSMUSG00000026896	Ifih1	1235.73	581.23	-1.09	0.010587	Down
ENSMUSG00000031328	Flna	8909.97	6515.71	-0.45	0.01076	Down
ENSMUSG00000032089	Il10ra	718.25	349.24	-1.04	0.011412	Down

ENSMUSG00000029570	Lfng	603.15	357.85	-0.75	0.011468	Down
ENSMUSG00000033392	Clasp2	898.89	690.13	-0.38	0.011888	Down
ENSMUSG00000026042	Col5a2	1849.64	1059.46	-0.80	0.012148	Down
ENSMUSG00000033777	Tlr13	311.71	155.50	-1.00	0.012541	Down
ENSMUSG00000028864	Hgf	156.31	94.48	-0.73	0.012581	Down
ENSMUSG00000039994	Timeless	316.48	218.29	-0.54	0.013199	Down
ENSMUSG00000031709	Tbc1d9	669.59	455.73	-0.56	0.013582	Down
ENSMUSG00000079445	B3gnt7	185.66	116.86	-0.67	0.014186	Down
ENSMUSG00000008398	Elk3	877.80	643.01	-0.45	0.01451	Down
ENSMUSG00000035547	Capn5	1014.17	714.09	-0.51	0.01451	Down
ENSMUSG00000029923	Rab19	176.66	85.31	-1.05	0.014702	Down
ENSMUSG00000022048	Dpysl2	525.75	346.52	-0.60	0.015116	Down
ENSMUSG00000028954	Nub1	1433.59	1024.14	-0.49	0.015309	Down
ENSMUSG00000018340	Anxa6	3451.51	2577.65	-0.42	0.015986	Down
ENSMUSG00000020357	Flt4	801.29	529.88	-0.60	0.016419	Down
ENSMUSG00000051236	Msrb3	487.95	342.12	-0.51	0.016501	Down
ENSMUSG00000024696	Lpxn	194.22	95.63	-1.02	0.016854	Down
ENSMUSG00000055401	Fbxo6	1707.90	1134.83	-0.59	0.01773	Down
ENSMUSG00000019139	Isyna1	2684.04	2141.19	-0.33	0.017733	Down
ENSMUSG00000052889	Prkcb	148.14	77.62	-0.93	0.017959	Down
ENSMUSG00000015143	Actn1	6485.14	4073.28	-0.67	0.018001	Down
ENSMUSG00000024789	Jak2	2754.56	1847.59	-0.58	0.018275	Down
ENSMUSG00000062300	Pvrl2	2219.13	1700.42	-0.38	0.018497	Down
ENSMUSG00000051379	Flrt3	134.98	76.04	-0.83	0.018622	Down

ENSMUSG00000031799	Tpm4	6271.05	4774.16	-0.39	0.018692	Down
ENSMUSG00000059142	Zfp945	640.67	468.57	-0.45	0.019516	Down
ENSMUSG00000006360	Crip1	859.80	509.05	-0.76	0.019526	Down
ENSMUSG00000026102	Inpp1	443.79	254.35	-0.80	0.019526	Down
ENSMUSG00000039981	Zc3h12d	127.33	53.18	-1.26	0.01992	Down
ENSMUSG00000024610	Cd74	78002.64	47007.99	-0.73	0.020321	Down
ENSMUSG00000027009	Itga4	406.14	175.06	-1.21	0.020475	Down
ENSMUSG00000025203	Scd2	3010.05	2316.31	-0.38	0.020479	Down
ENSMUSG00000074622	Mafb	707.83	496.60	-0.51	0.020479	Down
ENSMUSG00000020275	Rel	74.41	33.04	-1.17	0.021287	Down
ENSMUSG00000031627	Irf2	1243.09	825.04	-0.59	0.021514	Down
ENSMUSG00000000127	Fer	356.65	243.70	-0.55	0.021595	Down
ENSMUSG00000026728	Vim	5863.78	3413.38	-0.78	0.022117	Down
ENSMUSG00000079057	Cyp4v3	694.58	459.53	-0.60	0.022118	Down
ENSMUSG00000079442	St6galnac4	281.15	164.59	-0.77	0.022392	Down
ENSMUSG00000001156	Mxd1	607.24	386.16	-0.65	0.023326	Down
ENSMUSG00000021250	Fos	356.68	165.59	-1.11	0.023366	Down
ENSMUSG00000024910	Ctsw	128.61	63.88	-1.01	0.023395	Down
ENSMUSG00000039264	Gimap3	88.20	43.16	-1.03	0.023718	Down
ENSMUSG00000038884	A230050P20Rik	399.06	257.19	-0.63	0.024137	Down
ENSMUSG00000023927	Satb1	117.48	62.59	-0.91	0.024161	Down
ENSMUSG00000085887	Arhgap27os3	50.82	20.49	-1.31	0.025338	Down
ENSMUSG00000040711	Sh3pxd2b	589.29	378.85	-0.64	0.025348	Down
ENSMUSG00000026094	Stk17b	810.96	570.95	-0.51	0.025566	Down

ENSMUSG00000040809	Chil3	97.93	22.70	-2.11	0.025904	Down
ENSMUSG00000020092	Pald1	489.64	357.73	-0.45	0.025973	Down
ENSMUSG00000001444	Tbx21	56.48	17.91	-1.66	0.02647	Down
ENSMUSG00000024556	Me2	406.63	284.67	-0.51	0.02647	Down
ENSMUSG00000000204	Slfn4	198.59	86.30	-1.20	0.026898	Down
ENSMUSG00000021262	Evl	540.35	365.58	-0.56	0.02764	Down
ENSMUSG00000049892	Rasd1	677.45	248.73	-1.45	0.028333	Down
ENSMUSG00000042842	Serpinb6b	2244.58	1455.88	-0.62	0.028781	Down
ENSMUSG00000065954	Tacc1	1922.53	1459.17	-0.40	0.029153	Down
ENSMUSG00000015653	Steap2	4347.98	3066.06	-0.50	0.029354	Down
ENSMUSG00000062488	I830012O16Rik	742.36	302.68	-1.29	0.029354	Down
ENSMUSG00000028364	Tnc	1257.55	639.68	-0.98	0.0297	Down
ENSMUSG00000031948	Kars	3226.42	2245.09	-0.52	0.029945	Down
ENSMUSG00000081665	Gm15922	61.67	19.69	-1.65	0.030124	Down
ENSMUSG00000059248	9-Sep	2378.20	1817.00	-0.39	0.030953	Down
ENSMUSG00000032344	Mb21d1	106.18	58.42	-0.86	0.031826	Down
ENSMUSG00000030107	Usp18	841.68	191.91	-2.13	0.031856	Down
ENSMUSG00000052160	Pld4	1116.13	654.51	-0.77	0.031884	Down
ENSMUSG00000097804	Gm16685	112.48	53.66	-1.07	0.032704	Down
ENSMUSG00000006403	Adamts4	599.85	255.10	-1.23	0.032791	Down
ENSMUSG00000042404	Dennd4b	387.72	275.77	-0.49	0.032792	Down
ENSMUSG00000020134	Peli1	729.30	510.42	-0.51	0.032852	Down
ENSMUSG00000009585	Apobec3	485.76	304.05	-0.68	0.032922	Down
ENSMUSG00000020589	Fam49a	403.47	251.05	-0.68	0.033875	Down

ENSMUSG00000051344	Plekhm3	524.77	378.61	-0.47	0.034084	Down
ENSMUSG00000032193	Ldlr	532.56	282.18	-0.92	0.034705	Down
ENSMUSG00000001995	Sipa1l2	666.01	441.51	-0.59	0.034708	Down
ENSMUSG00000000693	Loxl3	288.31	188.23	-0.62	0.034783	Down
ENSMUSG00000013584	Aldh1a2	1319.16	816.48	-0.69	0.035085	Down
ENSMUSG00000021377	Dek	2326.48	1874.33	-0.31	0.035586	Down
ENSMUSG00000032091	Tmprss4	93.99	37.46	-1.33	0.036359	Down
ENSMUSG00000042350	Arel1	1365.13	934.79	-0.55	0.036488	Down
ENSMUSG00000022952	Runx1	1265.03	715.72	-0.82	0.036542	Down
ENSMUSG00000020901	Pik3r5	290.70	165.90	-0.81	0.037067	Down
ENSMUSG00000008496	Pou2f2	233.62	149.56	-0.64	0.037316	Down
ENSMUSG00000027797	Dclk1	191.42	123.94	-0.63	0.037533	Down
ENSMUSG00000027540	Ptpn1	2677.99	1910.68	-0.49	0.037642	Down
ENSMUSG00000041135	Ripk2	504.01	326.13	-0.63	0.039524	Down
ENSMUSG00000026976	Pax8	3322.10	2379.66	-0.48	0.039652	Down
ENSMUSG00000004099	Dnmt1	1372.16	1092.98	-0.33	0.039727	Down
ENSMUSG00000056427	Slit3	376.20	233.59	-0.69	0.040023	Down
ENSMUSG00000015217	Hmgb3	2611.29	2009.89	-0.38	0.04028	Down
ENSMUSG00000038372	Gmds	889.34	627.85	-0.50	0.04028	Down
ENSMUSG00000075225	Ccdc162	93.98	53.27	-0.82	0.040924	Down
ENSMUSG00000024457	Trim26	1553.59	1163.84	-0.42	0.041378	Down
ENSMUSG00000028693	Nasp	1079.30	868.61	-0.31	0.041905	Down
ENSMUSG00000059743	Fdps	877.60	583.76	-0.59	0.04401	Down
ENSMUSG00000054405	Dnajc8	1627.07	1342.01	-0.28	0.044693	Down

ENSMUSG00000026773	Pfkfb3	1067.97	586.38	-0.86	0.045688	Down
ENSMUSG00000024981	Acsl5	3363.28	2540.54	-0.40	0.04704	Down
ENSMUSG00000038811	Gngt2	236.27	144.28	-0.71	0.047638	Down
ENSMUSG00000024300	Myo1f	636.97	304.86	-1.06	0.049133	Down
ENSMUSG00000039115	Itga9	943.40	665.63	-0.50	0.049367	Down
ENSMUSG00000031278	Acsl4	3467.69	2114.11	-0.71	0.049392	Down
ENSMUSG00000070407	Hs3st3b1	188.13	108.21	-0.80	0.049655	Down
ENSMUSG00000056394	Lig1	596.69	433.96	-0.46	0.049782	Down

Supplementary Table 3. qPCR primers list

Gene Name	TaqMan Probe	Species
Apex1	Mm01319526_g1	Mouse
Acta2	Mm00725412_s1	Mouse
Casp3	Mm01195085_m1	Mouse
Ccl2	Mm00441242_m1	Mouse
Col1a1	Mm00801666_g1	Mouse
Ctgf	Mm01192933_g1	Mouse
Fn1	Mm01256744_m1	Mouse
Gapdh	Mm99999915_g1	Mouse
Havcr1	Mm00506686_m1	Mouse
Hmox1	Mm00516005_m1	Mouse
Infg	Mm01168134_m1	Mouse
Il1b	Mm00434228_m1	Mouse
Il6	Mm00446190_m1	Mouse
Il10	Mm01288386_m1	Mouse
Lcn2	Mm01324470_m1	Mouse
Nos2	Mm00440502_m1	Mouse
Ptgs2	Mm00478374_m1	Mouse
Spp1	Mm00436767_m1	Mouse
Tnf	Mm00443258_m1	Mouse
Trp53	Mm01731290_g1	Mouse
